# Real‐world efficacy and safety outcomes of imatinib treatment in patients with chronic myeloid leukemia: An Australian experience

**DOI:** 10.1002/prp2.1005

**Published:** 2022-09-14

**Authors:** Josephine A. Adattini, Annette S. Gross, Nicole Wong Doo, Andrew J. McLachlan

**Affiliations:** ^1^ Sydney Pharmacy School The University of Sydney Sydney New South Wales Australia; ^2^ Clinical Pharmacology Modelling & Simulation GlaxoSmithKline R &D Sydney New South Wales Australia; ^3^ Concord Cancer Centre Concord Repatriation General Hospital Sydney New South Wales Australia

**Keywords:** anticancer drugs, chronic myeloid leukemia, imatinib, pharmacoepidemiology, precision medicine, real‐world evidence, tyrosine kinase inhibitors

## Abstract

Tyrosine kinase inhibitors (TKI) have revolutionized the treatment of chronic myeloid leukemia (CML), but patients still experience treatment‐limiting toxicities or therapeutic failure. To investigate the real‐world use and outcomes of imatinib in patients with CML in Australia, a retrospective cohort study of patients with CML commencing imatinib (2001–2018) was conducted across two sites. Prescribing patterns, tolerability outcomes, and survival and molecular response were evaluated. 86 patients received 89 imatinib treatments. Dose modifications were frequently observed (12‐month rate of 58%). At last follow‐up, 62 patients (5‐year rate of 55%) had permanently discontinued imatinib treatment, of which 44 switched to another TKI (5‐year rate of 46%). Within 3 months of starting imatinib, 43% (95% CI, 32%–53%) of patients experienced imatinib‐related grade ≥3 adverse drug reactions (ADRs). Higher comorbidity score, lower body weight, higher imatinib starting dose, and Middle Eastern or North African ancestry were associated with a higher risk of grade ≥3 ADR occurrence on multivariable analysis (MVA). Estimated overall survival and event‐free survival rates at 3 years were 97% (95% CI, 92%–100%) and 81% (95% CI, 72%–92%), respectively. Cumulative incidence of major molecular response (MMR) at 3 years was 63% (95% CI, 50%–73%). On MVA, imatinib starting dose, ELTS score, *BCR‐ABL1* transcript type, pre‐existing pulmonary disease, and potential drug–drug interactions were predictive of MMR. In conclusion, imatinib induced deep molecular responses that translated to good survival outcomes in a real‐world setting, but was associated with a higher incidence of ADRs, dose modifications and treatment discontinuations than in clinical trials.

## Graphical abstract

This study found that imatinib induces fast and deep molecular responses that translate to good survival outcomes in a real‐world setting, however a high frequency of grade ≥ 3 adverse drug reactions (ADRs) and treatment‐limiting ADRs were observed.

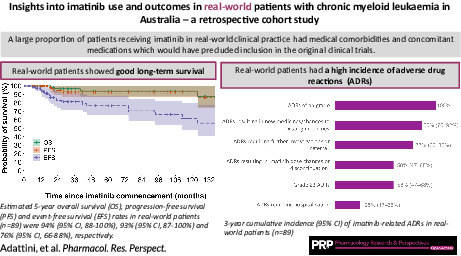

**Abbreviations**
ADRs: adverse drug reactions
AML: acute myeloid leukemia
BCRP: breast cancer resistance protein
BM: bone marrow
C_ss,min_
: rough plasma concentrations at steady state
CAM: complementary or alternative medicine
CCI: Charlson Comorbidity Index
CCyR: complete cytogenetic response
CI: confidence interval
CML: chronic myeloid leukemia
CTCAE v5: common terminology criteria for adverse events version 5.0
CYP: cytochrome P450
DMR: deep molecular response
ECOG: Eastern Cooperative Oncology Group
EFS: event‐free survival
ELTS: European Treatment and Outcome Study (EUTOS) long‐term survival
EMR: early molecular response
HR: hazard ratio
IQR: interquartile range
MMR: major molecular response
MR: molecular response
MVA: multivariable analysis
OR: odds ratio
OS: overall survival
PBPK: physiologically based pharmacokinetic
PD: pharmacodynamic
PFS: progression‐free survival
P‐gp: P‐glycoprotein
PK: pharmacokinetic
PS: performance status
SD: standard deviation
sDMR: sustained deep molecular response
SHR: subdistribution hazard ratio
TFR: treatment‐free remission
TKI: Tyrosine kinase inhibitors
TTD: time to discontinuation
TTNT: time to next line of treatment

## 1 INTRODUCTION

Imatinib, a *BCR‐* *ABL1* tyrosine kinase inhibitor (TKI), has significantly changed the treatment landscape of chronic myeloid leukemia (CML). In the landmark IRIS trial, imatinib induced complete cytogenetic response (CCyR) in 85.2% of patients by 18 months, compared to only 22.1% with interferon‐α plus low‐dose cytarabine, and was also better tolerated with significantly superior survival outcomes.^1^ Despite widespread introduction of second and third‐generation TKIs, imatinib used in first‐line is associated with a lower incidence of adverse drug reactions (ADRs) and similar long‐term survival outcomes.^2^

A significant proportion of patients receiving imatinib for CML management do not achieve major molecular response (MMR) or deep molecular response (DMR) on long‐term treatment (5‐year cumulative incidence of 60% and 42%, respectively^3^), whilst others develop resistance to treatment or intolerable ADRs necessitating treatment discontinuation.^3^, ^4^ About one‐third of patients achieve sustained deep molecular response (sDMR, 8‐year cumulative incidence of 37%^5^); considered the gateway to obtaining treatment‐free remission (TFR).

A precision medicine approach is required to improve the utilization of this lifesaving drug, to reduce the risk of ADRs and treatment failure, to restore and maintain good health‐related quality of life, and potentially to achieve a cure at an affordable cost.^6^ It is important to understand the gap between patients enrolled in clinical trials and the real‐world setting, with only 6% of patients diagnosed with a new cancer between 2018 and 2019 in Australia participating in a clinical trial.^7^ The FDA recently published a framework highlighting the importance of using real‐world observational data as a supplement to clinical trial data, to provide a more complete picture of tolerability and effectiveness of a drug.^8^ Clinical trials employ strict patient inclusion and exclusion criteria, which can lead to populations in clinical trials that differ significantly from patients found in real‐world clinical practice.^9^, ^10^ A review of eligibility criteria for cancer clinical trials submitted as investigational new drug applications to the FDA in 2015 found that 74% of trials excluded patients with a history of cardiovascular disease, 70% excluded patients with known hepatitis, 32% excluded patients with autoimmune diseases, and 29% excluded patients with gastrointestinal disorders.^11^ Clinical trials also exclude patients on certain concomitant medicines for chronic health conditions, which have the potential for pharmacokinetic (PK) or pharmacodynamic (PD) drug–drug interactions with the investigational drug. Furthermore, approximately 60% of oncology clinical trials require an Eastern Cooperative Oncology Group (ECOG) performance status (PS) of 0 or 1 (or an equivalent Karnofsky PS of ≥70%), resulting in the exclusion of patients with poorer prognosis.^11^ These exclusion criteria indirectly result in significant age disparities and differences in concomitant medication burden compared to patients who will ultimately receive the drug in practice.^12^ Therefore, there is uncertainty as to the extent which findings from oncology clinical trials can be extrapolated (or generalized) to the heterogenous population of patients with comorbid conditions that are treated in real‐world routine clinical practice.^10^

The aim of this study was to investigate the real‐world use, tolerability, and efficacy outcomes of imatinib in patients with CML treated in Australian hematology clinical practice.

## 2 MATERIALS AND METHODS

### 2.1 Study design

A retrospective observational study of patients with CML commencing imatinib between 2001–2018 was conducted at two University teaching hospitals in Sydney, Australia. Patients were identified through department registries and physician lists and were excluded if medical records were incomplete or if they had not received at least 3 months of TKI treatment. Data were collected on each patient from the first documented physician visit through to the date of last documented physician review by the data collection cut‐off date (November 2018), next TKI commencement, or death, whichever came first. The study was conducted in accordance with ethics requirements of local institutions (Appendix S1, Supplementary Methods).

### 2.2 Data collection

Individual medical records were retrospectively reviewed, and demographic characteristics, disease characteristics, treatment details, prescribing patterns, tolerability outcomes, and efficacy outcomes were collected. Data were re‐abstracted and verified by a second investigator in 30% of randomly selected patients. Variables and endpoints are defined in Appendix S1, Supplementary Methods.

Demographic characteristics included geographic ancestry, comorbidities, kidney and hepatic function, age, sex, and total body weight at the time of CML diagnosis and at imatinib commencement. Geographic ancestry was assigned based on information contained on patient registration forms and in the medical record, taking into consideration documented self‐reported geographic ancestry or inference of geographic ancestry (using birthplace, family name or maiden name, language[s] spoken, and religion).^13^, ^14^, ^15^ The Charlson Comorbidity Index (CCI) score was derived from comorbidities noted in patients' medical records and included their diagnosis of CML.^16^ Any concomitant medicines that the patient used regularly during their TKI treatment (for ≥2 weeks) were documented, including recorded complementary and alternative medicines. Disease‐related variables collected included ECOG PS,^17^ CML disease phase, presence of bone marrow (BM) fibrosis, BM karyotype abnormalities in addition to the t(9;22)(q34;q11), and *BCR‐ABL1* transcript type at diagnosis.^18^, ^19^ Sokal^20^ and European Treatment and Outcome Study (EUTOS) long‐term survival (ELTS)^21^ risk scores at CML diagnosis were calculated.

Treatment‐related variables collected included index date (date of first imatinib prescription), starting dose, and line of treatment (first‐line vs. second‐line or later). Details of any imatinib dose modifications (dose escalations, dose reductions, changes in dose frequency, or temporary treatment interruptions) were collected, including the date of dose modification, reason(s), and immediate consequence(s). Permanent discontinuation of imatinib treatment was documented, in addition to the reasons for discontinuation. This data were used to calculate the following endpoints: time to first dose modification, time to discontinuation (TTD), and time to next line of treatment (TTNT).

All documented adverse events during imatinib treatment were evaluated for causality to imatinib using the Naranjo algorithm.^22^ Adverse events classified as possible, probable, and definite were termed as imatinib‐related ADRs.^23^ The type and severity grade of adverse events were defined using the Common Terminology Criteria for Adverse Events version 5.0 (CTCAE v5).^24^ The time to occurrence and management of adverse events (i.e. dose modification, hospitalization) were also recorded.

Molecular response (MR) endpoints were defined using quantitative *BCR‐ABL1* transcript levels. *BCR‐ABL1* transcript levels of ≤0.1%, ≤0.01%, ≤0.0032%, ≤0.001% on the international scale (IS) were defined MMR, MR^4.0^, MR^4.5^, and MR^5.0^ respectively.^18^ MR^4.0^, MR^4.5^, and MR^5.0^ are classified as DMR.^18^ Achievement of sDMR (DMR maintained for at least 2 consecutive years) and early molecular response (EMR; *BCR‐ABL1* ^*IS*^ ≤ 10% at 3 and 6 months) were also documented. Survival endpoints included overall survival (OS), progression‐free survival (PFS), and event‐free survival (EFS).^18^ OS was calculated from the date of imatinib initiation until death (of any cause, whilst on imatinib or within 60 days off imatinib treatment) or the end of treatment follow‐up, whichever occurred earliest. PFS was calculated from the date of imatinib initiation until disease progression to accelerated/blast phase, transformation to acute myeloid leukemia (AML), or death (of any cause), while on imatinib or within 60 days off imatinib treatment. EFS was defined as survival with the absence of disease progression, relapse, or death (of any cause), while receiving imatinib or within 60 days off imatinib treatment.

### 2.3 Statistical analysis

Continuous variables are presented as mean and standard deviation (SD), or median and interquartile range (IQR), and compared between groups using the independent two‐sample t‐test or the non‐parametric Wilcoxon‐Mann–Whitney test, respectively. Categorical variables were described using frequencies and percentages and compared between groups using Pearson's chi‐squared test of independence or Fisher's exact test of independence if Cochran's rule was not met.

Survival endpoints (OS, PFS, & EFS), time to first dose modification, TTD, and TTNT were evaluated using the Kaplan–Meier method, with a log‐rank test comparing between‐group differences.^25^ Patients without an event were censored at the date of last follow‐up. A Cox proportional hazards model^26^ was used to assess the independent factors associated with EFS. Data are reported as hazard ratios (HRs) with associated 95% confidence intervals (CIs).

A logistics regression model was used to investigate the effect of baseline variables on achievement of EMR at 3 months, with a Wald test to assess the null hypothesis of no between‐group difference.^27^ Odds ratios (ORs) and associated 95% CIs are reported.

The cumulative incidences of molecular response (MMR, DMR, sDMR) and imatinib‐related ADRs were modeled using the cumulative incidence competing risk method, with Gray's weighted log‐rank test comparing between‐group differences.^28^, ^29^, ^30^ Competing risks were treatment discontinuation or death prior to molecular response or occurrence of the ADR of interest. The cumulative incidence of molecular response was calculated among evaluable patients who had valid molecular monitoring at baseline and during TKI treatment. A Fine‐Gray subdistribution hazard model^31^, ^32^, ^33^, ^34^, ^35^ was used to assess the independent factors associated with MMR achievement and grade ≥ 3 ADR occurrence. Subdistribution hazard ratios (SHRs) and associated 95% CIs are reported. The hazard of recurrent ADRs was modeled using the Prentice, Williams, and Peterson total time model,^36^ with data presented as HRs and their 95% CIs.

To investigate the clinical outcomes for real‐world patients who would have been considered ineligible for a controlled clinical trial, subgroup analyses were performed based on an individual patient's likely eligibility for inclusion in the pivotal ENESTnd^37^ and DASISION^38^ trials. Clinical trial eligibility categorization (eligible vs. ineligible) considered an individual patient's ECOG PS, pre‐existing comorbidities and concomitant medicines, as per ENESTnd and DASISION clinical trial exclusion criteria (Appendix S1, Supplementary Methods, Table S1). Exclusion criteria known not to reflect risks with imatinib treatment (e.g. receiving concomitant treatment with QTc interval prolonging medicines) were not included in the clinical trial eligibility categorization in this study. ENESTnd and DASISION trials were chosen over the landmark IRIS trial^1^ and CML‐study IV^39^ to reflect current clinical practice where first‐line CML treatment is monotherapy with a tyrosine kinase inhibitor (i.e. nilotinib, dasatinib, imatinib).^40^ Additionally, unlike the IRIS trial and CML‐study IV, the ENESTnd trial considered potential PK and PD drug interactions with imatinib in its eligibility criteria (cytochrome P450 [CYP]3A4 inducers/inhibitors and anticoagulant use, respectively).^41^

A multiple imputation method using the Multivariate Imputation via Chained Equations package in R^42^ was used to impute values for variables with missing observations for use during multivariable regression analyses. The multiple imputation method assumes that data are missing at random and implements an iterative Markov chain Monte Carlo type of algorithm. Complete‐case analysis, in which only subjects with all values recorded for all covariates are retained in the analysis, was not used due to the possibility of introducing bias and producing estimates with higher variance.^43^, ^44^ Statistical methods for data analysis are further justified and defined in the Appendix S1, Supplementary Methods. All reported *p* values are two‐sided, and a significance level of *α* = .05 was used (except for selection of variables for inclusion in multivariable regression, whereby a significance level of *α* = .10 was used). The statistical analyses were performed using R (version 3.3.3).^45^

### 2.4 Nomenclature of targets and ligands

Key protein targets and ligands in this article are hyperlinked to corresponding entries in http://www.guidetopharmacology.org, the common portal for data from the IUPHAR/BPS Guide to PHARMACOLOGY,^46^ and are permanently archived in the Concise Guide to PHARMACOLOGY 2019/20.^47^

## 3 RESULTS

86 patients who initiated imatinib from 2001 to 2018 met eligibility and were included in the analysis. The median follow‐up time, from the index date to the earliest of death, change in treatment or last encounter in medical records, was 33 months (IQR 14 to 102 months). Baseline demographic and CML characteristics of all patients treated with imatinib (*n* = 86) are summarized in Table 1.

**TABLE 1:** Baseline demographic and CML disease characteristics of the imatinib treated cohort, including comparison by likely eligibility for the ENESTnd^37^ and DASISION^38^ trials

Characteristics	All imatinib‐treated patients	Eligibility for ENESTnd and DASISION		
		Eligible	Ineligible	*p* Value*
	(*N* = 86)	(*N* = 39)	(*N* = 47)	
Age at diagnosis (years), mean (SD)	55 (17)	44 (14)	64 (13)	<.001*
CCI score, median (range; IQR)	4 (2–12; 2.25–5)	2 (2–6; 2–3)	5 (2–12; 4–7)	<.001*
Male, *n* (%)	51 (59)	21 (54)	30 (64)	.34
Geographic ancestry, *n* (%)^a^				
European	64 (74)	28 (72)	36 (77)	.77
Other^b^	6 (7)	2 (5)	4 (9)	
South Asian	4 (5)	2 (5)	2 (4)	
Other^b^	6 (7)	2 (5)	4 (9)	
Comorbidities at diagnosis, *n* (%)				
Cardiovascular disease	21 (24)	0	21 (45)	<.001*
Poorly controlled diabetes	13 (15)	0	13 (28)	<.001*
Poorly controlled hypertension	12 (14)	0	12 (26)	<.001*
Chronic pulmonary disease	11 (13)	0	11 (23)	<.001*
Peripheral vascular disease	10 (12)	0	10 (21)	<.05*
Hypothyroidism post thyroidectomy	4 (5)	0	4 (9)	.13
History of pancreatitis	2 (2)	0	2 (4)	.50
Cerebrovascular disease	2 (2)	0	2 (4)	.50
None of the above	42 (49)	39 (100)	3 (6)	<.05*
Family history of cardiovascular disease, *n* (%)				
Yes	25 (52)	10 (48)	15 (56)	.59
No	23 (48)	11 (52)	12 (44)	
Unknown	38	18	20	
Concomitant medicines, *n* (%)				
CYP3A4 substrate	45 (52)	16 (41)	29 (62)	.06
Antiplatelet	40 (47)	8 (21)	32 (68)	<.001*
Paracetamol	10 (12)	2 (5)	8 (17)	.09
Antineoplastic	9 (10)	2 (5)	7 (15)	.17
Digoxin	7 (8)	0	7 (15)	<.05*
Thyroxine	6 (7)	0	6 (13)	<.05*
CYP2C8 inhibitor	6 (7)	0	6 (13)	<.05*
P‐gp inhibitor	5 (6)	0	5 (11)	.06
CYP3A4 inhibitor	4 (5)	0	4 (9)	<.05*
CYP3A4 inhibitor, CAM	3 (4)	0	3 (6)	
CYP3A4 inducer	1 (1)	0	1 (2)	.50
CYP3A4 inducer, CAM	1 (1)	0	1 (2)	
None of the above	25 (29)	19 (49)	6 (13)	<.001*
Disease phase, *n* (%)				
Chronic	78 (91)	35 (90)	43 (91)	.78
Accelerated	8 (9)	4 (10)	4 (9)	
Extramedullary leukemia present, *n* (%)	1 (1)	0	1 (2)	1
ECOG PS, *n* (%)				
ECOG PS 0	52 (60)	26 (67)	26 (55)	.54^c^
ECOG PS 1	29 (34)	12 (31)	17 (36)	
ECOG PS 2	4 (5)	1 (3)	3 (6)	
ECOG PS 3	0	0	0	
ECOG PS 4	1 (1)	0	1 (2)	
Sokal score, *n* (%)				
Low	19 (24)	10 (29)	9 (20)	.66
Intermediate	40 (50)	16 (46)	24 (53)	
High	21 (26)	9 (26)	12 (27)	
Unknown	6	4	2	
ELTS score, *n* (%)				
Low	40 (50)	22 (63)	18 (40)	.12^d^
Intermediate	26 (33)	9 (26)	17 (38)	
High	14 (18)	4 (11)	10 (22)	
Unknown	6	4	2	
Additional BM karyotype abnormalities, *n* (%)				
Yes	6 (10)	5 (17)	1 (3)	.10
No	54 (90)	24 (83)	30 (97)	
Unknown	26	10	16	
BM fibrosis, *n* (%)				
Yes	35 (76)	14 (78)	21 (75)	1
No	11 (24)	4 (22)	7 (25)	
Unknown	40	21	19	
*BCR‐ABL1* transcript type, *n* (%)				
e13a2 (b2a2)	27 (42)	9 (33)	18 (49)	.45^e^
e14a2 (b3a2)	16 (25)	9 (33)	7 (19)	
e13a2 (b2a2) and e14a2 (b3a2)	7 (11)	4 (15)	3 (8)	
e14a2 (b3a2) and e1a2	6 (9)	3 (11)	3 (8)	
e13a2 (b2a2) and e1a2	4 (6)	2 (7)	2 (5)	
e1a2	2 (3)	0	2 (5)	
e19a2	1 (2)	0	1 (3)	
e12a2, e14a2 (b3a2) and e1a2	1 (2)	0	1 (3)	
Unknown	22	12	10	

Abbreviations: BM, bone marrow; CAM, complementary or alternative medicine; CCI, Charlson Comorbidity Index; CYP, cytochrome P450; ECOG PS, Eastern Cooperative Oncology Group Performance Status; ELTS, European Treatment and Outcome Study (EUTOS) long‐term survival; IQR, interquartile range; P‐gp, P‐glycoprotein; SD, standard deviation.

^a^ Geographic ancestry was assigned using information contained on patient registration forms and in medical records.

^b^ 3 of Middle Eastern/North African ancestry (1 Lebanon, 1 Iran, 1 Egypt) and 3 of Pacific Islander (Maori) ancestry.

^c^ Difference between groups also not statistically significant if comparing ECOG PS of 0, 1 and 2 or more (*p* = 1).

^d^ Significant difference in ELTS risk between groups if comparing low versus intermediate to high‐risk (*p* < .05).

^e^ Comparison between e13a2, e14a2, e13a2 with e14a2, and other.

* Statistically significant difference (*α* < .05). Quantitative variables evaluated using the independent two‐sample *t*‐test or Wilcoxon‐Mann–Whitney test. Categorical variables evaluated using Pearson's chi‐squared test or Fisher's exact test.

### 3.1 Imatinib prescribing patterns

Of the 86 patients treated with imatinib, data were collected on a total of 89 imatinib treatment courses. In the majority of treatments (*n* = 78, 88%), imatinib was prescribed as first‐line therapy for newly diagnosed CML. Baseline demographic characteristics and distribution of disease risk scores were well‐balanced in patients receiving imatinib first‐line and those initiating imatinib second‐line or later (Table S2). Of patients receiving imatinib as first‐line treatment, 49% were initiated on the standard dose of imatinib 400 mg/day and 49% on a higher dose of 600 mg/day. In patients receiving imatinib treatment second‐line or later, the majority were initiated on 400 mg/day (64%) with only 27% initiated on 600 mg/day. Very few (*n* = 5, 6%) received imatinib with concomitant cancer treatments (e.g. peginterferon alfa‐2a, cytarabine, or hydroxyurea).

### 3.2 Imatinib dose modifications and treatment discontinuations

**FIGURE 1:**
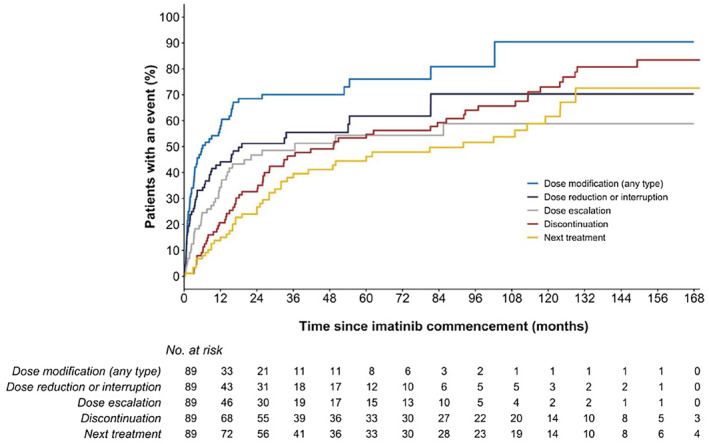
Kaplan–Meier estimated time to first imatinib dose modification (any type, dose reduction or treatment interruption, and dose escalation), time to imatinib discontinuation and time to next treatment.

Imatinib dose modifications were commonly observed in this real‐world cohort. Within the first 12 months of imatinib treatment, 58% (95% CI, 46% to 67%) of patients required an imatinib dose modification (of any type), 44% (95% CI, 33% to 54%) a dose reduction or temporary interruption of imatinib treatment, and 34% (95% CI, 23% to 44%) a dose escalation (Figure 1). Of patients requiring an imatinib dose reduction or temporary treatment interruption, 63% were receiving imatinib doses of 600 mg/day or greater at first dose change, whilst 35% were receiving 400 mg/day. Adverse events were the most common reason reported for imatinib dose reductions and treatment interruptions (86% of all dose reductions/interruptions). Of those requiring an imatinib dose escalation, 58% were initiated on a standard imatinib dose of 400 mg/day and 35% initiated on 600 mg/day. The most common reported reason(s) for dose escalation was poor response (54% of all dose escalations), followed by good tolerability (43%), and relapse or disease progression (8%).

At last follow‐up, only 30% of patients were still receiving imatinib treatment (26% of first‐line treatments and 64% of second‐line or later treatments; *p* < .05), with a median TTD of 49 months (95% CI, 28 to 93 months). The probability of imatinib discontinuation within the first 12 months of imatinib treatment was 21% (95% CI, 12% to 29%), increasing to 34% (95% CI, 23% to 43%) by 2 years and 55% (95% CI, 42% to 65%) by 5 years (Figure 1). The most frequently reported reasons for imatinib discontinuation were adverse events (52% of imatinib discontinuations) and poor response (31%). Nine patients also discontinued imatinib treatment due to attainment of sDMR, of which two patients then relapsed within a median follow‐up period of 9 months (IQR 6 to 22) post discontinuation. Among those who discontinued imatinib treatment (*n* = 62), 44 patients (71%) switched to a second‐generation or third‐generation TKI for CML treatment, with a median TTNT of 92 months (95% CI, 41 to 124 months); 4 patients progressed to a hematopoietic stem cell transplant, 4 patients remained off treatment whilst maintaining molecular remission, 2 patients died, with the disposition of 8 patients unknown. The estimated probability of switching to a second or third‐generation TKI was 15% (95% CI, 7% to 22%) by 12 months of imatinib treatment, 27% (95% CI, 16% to 34%) by 2 years and 46% (95% CI, 33% to 57%) by 5 years (Figure 1).

### 3.3 Tolerability outcomes

**FIGURE 2:**
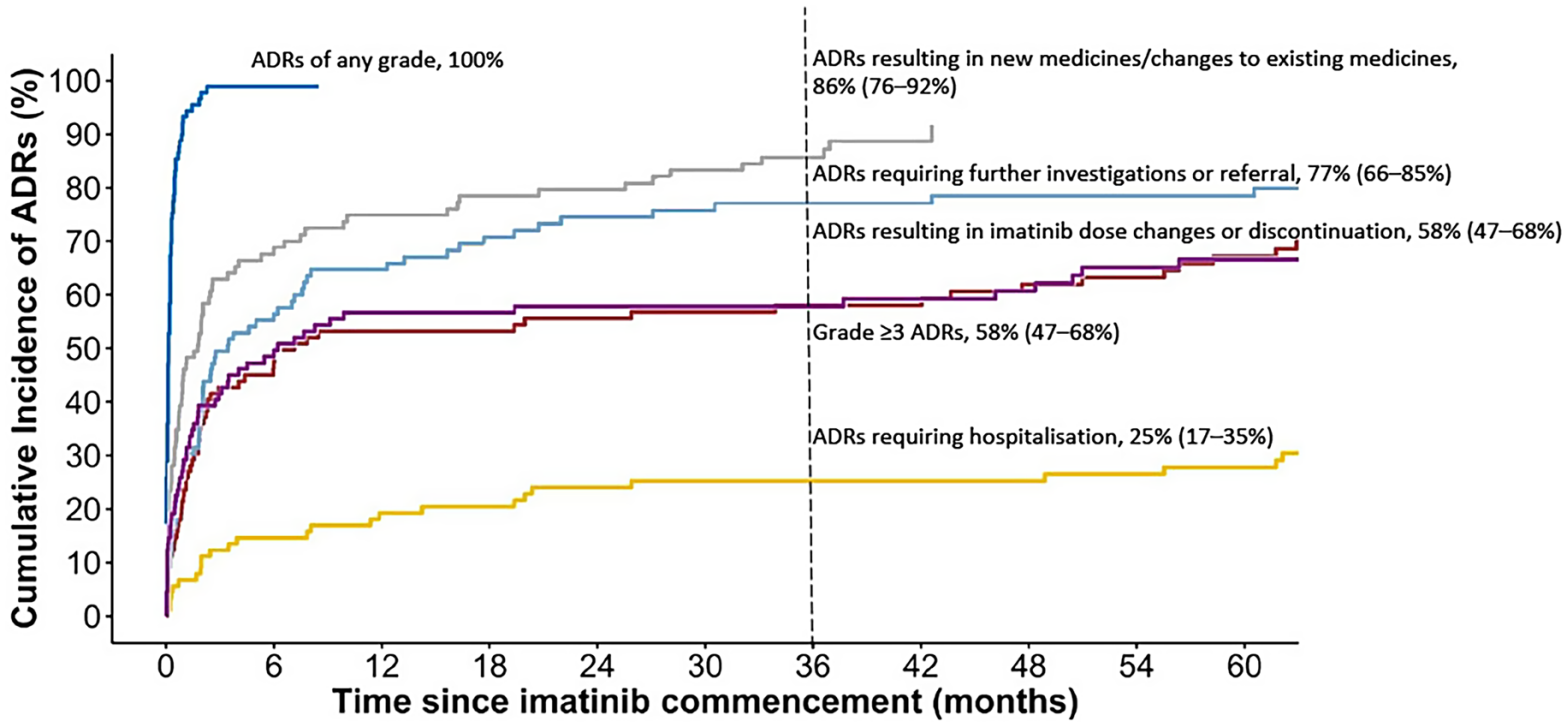
Cumulative incidence of imatinib‐related adverse drug reactions (ADRs) Cumulative incidence of imatinib‐related ADRs by 3 years (95% confidence intervals) calculated using the cumulative incidence competing risk method.

All patients experienced at least one imatinib‐related ADR during treatment (Table 2; Figure 2). The most frequent imatinib‐related ADRs of any grade included anemia, superficial oedema, leukopenia, neutropenia, thrombocytopenia, fatigue, muscle cramps, and infection (Table 3). Grade ≥ 3 imatinib‐related ADRs were common, with a 3‐month cumulative incidence of 43% (95% CI, 32% to 53%) and 18‐month cumulative incidence of 53% (95% CI, 42% to 63%; Table 2; Figure 2). The most frequent grade ≥ 3 imatinib‐related ADRs included neutropenia, leukopenia, rash, thrombocytopenia, anemia, hypertension or vascular disorders and superficial edema (Table 3).

**TABLE 2:** Cumulative incidence of imatinib‐related adverse drug reactions (ADRs), compared by likely eligibility for the ENESTnd^37^ and DASISION^38^ trials

Event	All imatinib treatments (*N* = 89)		Eligibility for ENESTnd and DASISION (ineligible [*N* = 48] versus eligible [*N* = 41])			
	Cumulative incidence at 3 months, % (95% CI)^a^	Cumulative incidence at 18 months, % (95% CI)^a^	SHR (95% CI) of an event^b^	*p* Value*	HR (95% CI) of event recurrence^c^	*p* Value*
Any ADR	99 (92–100)	100	0.94 (0.63–1.40)	0.76	1.19 (1.09–1.31)	<.001*
Hematological ADR or biochemical abnormality	83 (73–90)	93 (84–97)	1.13 (0.74–1.73)	0.56	1.16 (1.01–1.34)	<.05*
Non‐hematological ADR	92 (84–96)	98 (89–100)	1.14 (0.76–1.70)	0.53	1.23 (1.10–1.37)	<.001*
Any ADR, grade ≥ 3 (CTCAE v5)^d^	43 (32–53)	53 (42–63)	1.77 (1.08–2.91)	<0.05*	1.45 (1.05–2.01)	<.05*
Hematological ADR or biochemical abnormality, grade ≥ 3^d^	32 (22–41)	35 (25–45)	1.25 (0.65–2.42)	0.50	1.09 (0.69–1.71)	.71
Non‐hematological ADR, grade ≥ 3^d^	18 (11–27)	38 (28–48)	2.27 (1.29–4.00)	<0.05*	2.61 (1.66–4.10)	<.001*
ADR resulting in imatinib dose modification or treatment discontinuation	42 (31–52)	57 (46–66)	1.26 (0.75–2.10)	0.38	1.30 (0.99–1.70)	0.06
ADR resulting in commencement of medicines or changes to existing medicines^e^	63 (52–72)	78 (68–86)	1.24 (0.79–1.94)	0.35	1.44 (1.16–1.79)	<.05*
ADR resulting in hospitalization	12 (7–20)	20 (13–29)	2.36 (1.21–4.61)	<0.05*	1.89 (1.06–3.39)	<.05*
ADR requiring further investigations or referral to another healthcare professional	49 (39–59)	70 (59–78)	1.49 (0.93–2.39)	0.10	1.39 (1.05–1.83)	<.05*

Abbreviations: CI, confidence interval; HR, hazards ratio; SHR, subdistribution hazard ratio.

^a^ Cumulative incidences are calculated using the cumulative incidence competing risk method.

^b^ Subdistribution hazard ratios are calculated using the Fine‐Gray subdistribution hazards model. This represents the unadjusted hazard of the first event.

^c^ Hazard ratios of recurrent events are calculated using the Prentice, Williams and Peterson total time model. This represents the unadjusted hazard.

^d^ ADR severity classified using the National Cancer Institute Common Terminology Criteria for Adverse Events (CTCAE v5).

^e^ This included the commencement of short ‐term medicines for symptomatic management of ADR episodes (e.g. analgesics, antibiotics, antiemetics, antacids, diuretics, supplements to correct electrolyte imbalances and blood transfusions), dose changes to existing long‐term medicines or the commencement of new medicines to manage comorbidities arising from imatinib‐related ADRs (e.g. lipid lowering agents for hypercholesterolemia, thyroxine for hypothyroidism, antihypertensives and beta‐blockers for cardiovascular complications, antiplatelets or anticoagulants for treatment of embolic events, and inhalers for respiratory complications).

* Statistically significant difference (*α* < .05).

**TABLE 3:** Cumulative incidence of most common imatinib‐related adverse drug reactions

Adverse drug reaction (ADR) type (CTCAE v5)^a^	Cumulative incidence at 3 months, % (95% CI)^b^	Cumulative incidence at 18 months, % (95% CI)^b^
Top 20 most frequent ADRs of any grade		
Anemia	54 (43–64)	69 (58–78)
Superficial oedema	46 (35–56)	64 (53–74)
Leukopenia	52 (41–62)	60 (49–69)
Neutropenia	52 (41–62)	59 (48–68)
Thrombocytopenia	48 (38–58)	55 (44–65)
Fatigue	34 (24–44)	48 (27–58)
Muscle cramps	30 (21–40)	42 (31–52)
Infection	23 (14–32)	41 (30–51)
Diarrhea	20 (13–29)	40 (3–50)
Nausea	27 (18–37)	40 (29–50)
Rash	21 (14–30)	35 (25–45)
Creatinine elevation	23 (14–32)	30 (20–39)
Other hematological or biochemical ADRs	16 (9–24)	28 (19–38)
Hypocalcaemia	20 (13–29)	26 (17–36)
Hypophosphatemia	18 (11–27)	26 (17–36)
Arthralgia/arthritis	18 (11–27)	25 (16–34)
Other eye disorders	12 (7–20)	23 (15–32)
Vomiting	10 (5–18)	22 (14–31)
Weight gain	15 (8–23)	22 (14–31)
Pruritus	8 (3–15)	21 (13–30)
Top 10 most frequent grade ≥ 3 ADRs		
Neutropenia	18 (11–27)	18 (11–27)
Leukopenia	12 (7–20)	14 (7–22)
Rash	7 (3–13)	13 (7–20)
Thrombocytopenia	8 (3–15)	9 (4–16)
Anemia	3 (0.9–9)	6 (2–12)
Hypertension or other vascular disorders	1 (0.1–6)	5 (2–11)
Superficial oedema	1 (0.1–6)	5 (2–10)
Infection	1 (0.1–6)	4 (1–9)
Other hematological or biochemical ADRs	2 (0.4–7)	3 (1–9)
ALT elevation	2 (0.4–7)	3 (1–9)
Top 10 most frequent ADRs resulting in imatinib dose modifications or treatment discontinuation		
Nausea	7 (3–13)	16 (9–25)
Rash	8 (3–15)	15 (8–23)
Superficial oedema	7 (3–13)	15 (8–23)
Neutropenia	11 (6–19)	12 (7–20)
Thrombocytopenia	9 (4–16)	10 (5–18)
Leukopenia	8 (3–15)	9 (4–16)
Vomiting	2 (0.4–7)	8 (4–15)
Diarrhea	1 (0.1–6)	8 (4–15)
Anemia	5 (2–10)	8 (4–15)
Muscle cramps	2 (0.4–7)	6 (2–12)

Abbreviations: ALT, alanine transaminase; CI, confidence interval.

^a^ ADRs are classified using the National Cancer Institute Common Terminology Criteria for Adverse Events (CTCAE v5).

^b^ Cumulative incidences are calculated using the cumulative incidence competing risk method.

* Statistically significant difference (*α* < .05).

Overall, there was an incidence of 18.70 (95% CI, 15.25 to 23.30) imatinib‐related ADR episodes (of any grade) per 1‐person year of imatinib treatment, of which 1.50 (95% CI, 1.10 to 2.21) ADR episodes were grade ≥ 3 in severity. Medical management was required in 30% of imatinib‐related ADR episodes. By 18 months of imatinib treatment, 70% (95% CI, 58 to 78%) of patients had experienced at least one imatinib‐related ADR requiring further investigations or referral to another healthcare professional, 57% (95% CI, 46% to 66%) had experienced at least one ADR resulting in imatinib dose changes or discontinuation, and 20% (95% CI, 13% to 29%) had experienced an ADR resulting in hospitalization (Table 2). The most frequent ADRs resulting in imatinib dose modifications or discontinuation are presented in Table 3. Furthermore, 79% of patients (95% CI, 68% to 86%) had experienced at least one imatinib‐related ADR requiring commencement of short‐term medicines or changes in long‐term medicines (Table 2).

In univariable regression analysis, the following baseline variables were associated with a higher risk of occurrence of grade ≥ 3 ADRs with imatinib treatment: imatinib starting dose, total body weight, CCI score, concomitant use of a medicine with the potential for imatinib drug–drug interactions, treatment with another antineoplastic agent for CML, and family history of cardiovascular disease (*p* < .10; Table S3). In the multivariable model, an imatinib starting dose of 600 or 800 mg/day was independently predictive of a higher rate of occurrence of grade ≥ 3 ADRs at any time, compared to starting doses of 400 or 500 mg/day (SHR_adjusted_, 2.86; 95% CI, 1.49 to 5.56). A higher CCI score at diagnosis was independently associated with a higher hazard of grade ≥ 3 imatinib‐related ADRs (SHR_adjusted_, 1.20; 95% CI, 1.06 to 1.34). Additionally, a 10 kg decrease in total body weight was associated with a 25% increase in the hazard of grade ≥ 3 ADRs (SHR_adjusted_, 1.25; 95% CI, 1.04 to 1.45). Geographic ancestry was another significant predictor in the multivariable model, with patients of Middle Eastern or North African ancestry more likely to experience a grade ≥ 3 ADR during imatinib treatment compared to patients of European ancestry (SHR_adjusted_, 2.97; 95% CI, 1.22 to 7.20).

Predictors of recurrent grade ≥ 3 ADRs with imatinib treatment in univariable analysis included sex, age, total body weight, geographic ancestry, CCI score, ECOG PS, receiving a concomitant medicine with potential for imatinib drug–drug interactions, pre‐existing cardiovascular disease, pre‐existing pulmonary disease, pre‐existing peripheral vascular disease and a family history of cardiovascular disease (Table S3). In the multivariable model of recurrent events, patients commenced on an imatinib dose of 600 or 800 mg/day had a higher hazard of recurrent grade ≥ 3 ADRs at any time, compared to starting doses of 400 or 500 mg/day (HR_adjusted_, 1.49; 95% CI, 1.04 to 2.13). Receiving imatinib in combination with another anticancer agent for CML was associated with an increased risk of recurrent imatinib‐related grade ≥ 3 ADRs, compared to treatment with imatinib alone (HR_adjusted_, 2.18; 95% CI, 1.20 to 3.94). The multivariable model also indicated that female sex (HR_adjusted_, 1.72; 95% CI, 1.21 to 2.45), higher baseline CCI score (HR_adjusted_, 1.19; 95% CI, 1.10 to 1.28), and pre‐existing pulmonary disease (HR_adjusted_, 1.98 [95% CI, 1.26 to 3.12]) were independent predictors of recurrent imatinib‐related grade ≥ 3 ADRs. Finally, geographic ancestry was an important predictive factor for recurrent grade ≥ 3 ADRs on imatinib treatment (East Asians vs. Europeans HR_adjusted_, 1.93; 95% CI, 1.01 to 3.70).

### 3.4 Efficacy outcomes

Among patients evaluable for EMR (*n* = 60 at 3 months, *n* = 56 at 6 months), 73% achieved EMR at 3 months and 88% achieved EMR at 6 months of imatinib treatment. Univariable logistics regression identified that EMR achievement was significantly associated with imatinib starting dose, with a 5‐fold increase in the odds of achieving EMR in patients commenced on imatinib 600 or 800 mg/day compared to imatinib 400 or 500 mg/day (OR, 4.49; 95% CI, 1.13 to 22.99; *p* < .05). After adjusting for baseline patient characteristics (sex and total body weight), the likelihood of EMR in patients initiated on imatinib 600 or 800 mg/day remained significantly higher than those initiated on 400 or 500 mg/day (OR_adjusted_, 4.76; 95% CI, 1.03 to 25.00; *p* < .05).

**FIGURE 3:**
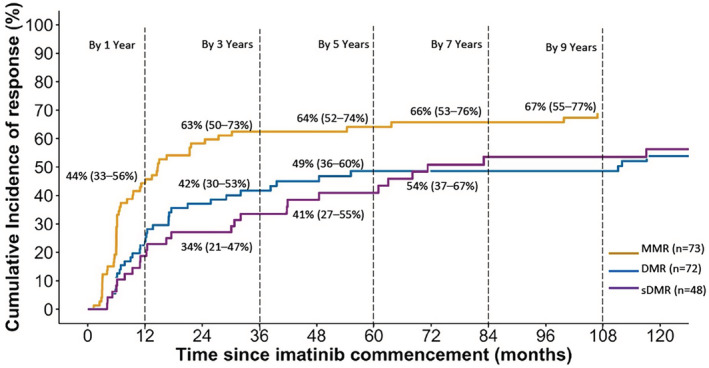
Cumulative incidence of major molecular response (MMR), deep molecular response (DMR) and sustained DMR (sDMR) in patients treated with imatinib. Cumulative incidence of molecular response at certain time points are presented with their associated 95% confidence intervals. Cumulative incidence was calculated using the cumulative incidence competing risk method.

The cumulative incidence of MMR among evaluable patients (*n* = 73) was 58% (95% CI, 46% to 69%) by 2 years, whilst the cumulative incidence of DMR (*n* = 72 evaluable) was 42% (95% CI, 30% to 53%) after 3 years and 49% (95% CI, 36% to 60%) after 5 years of imatinib treatment (Figure 3). A total of 48 patients had received imatinib treatment for at least 2 years with molecular monitoring sensitive enough to detect DMR, and hence were evaluable for sDMR. Of these, 26 patients (54%) achieved sDMR and thus were considered as potential candidates for drug discontinuation. The cumulative incidence of sDMR with imatinib treatment was 34% (95% CI, 21% to 47%) by 3 years and 41% (95% CI, 27% to 55%) by 5 years (Figure 3).

In univariable regression analysis, the following baseline variables were associated with MMR achievement; ELTS score, Sokal score, *BCR‐ABL1* transcript type and concomitant use of medicines with potential for imatinib drug–drug interactions (Table S4). In the multivariable model, an e14a2 *BRC‐ABL1* transcript type (vs. e13a2) and an intermediate or high ELTS score at diagnosis were both independent predictors of inferior MMR at any time during imatinib treatment (SHR, 0.46 [95% CI, 0.22 to 0.99], 0.41 [95% CI, 0.18 to 0.93] and 0.08 [95% CI, 0.02 to 0.36], respectively). Pre‐existing pulmonary disease was also predictive of a lower likelihood of MMR with imatinib treatment (SHR, 0.38; 95% CI, 0.17 to 0.85). Interestingly, concomitant use of a medicine with the potential for PK or PD drug–drug interactions with imatinib was predictive of higher MMR rates, compared to patients not receiving potentially interacting medicines (SHR, 3.27; 95% CI, 1.56 to 6.86). Finally, an imatinib starting dose of 400 or 500 mg/day was independently predictive of poorer MMR rates with imatinib treatment, compared to 600 or 800 mg/day (SHR, 0.33; 95% CI, 0.16 to 0.70). No other variables were found to be predictive in the multivariable regression analyses.

**FIGURE 4:**
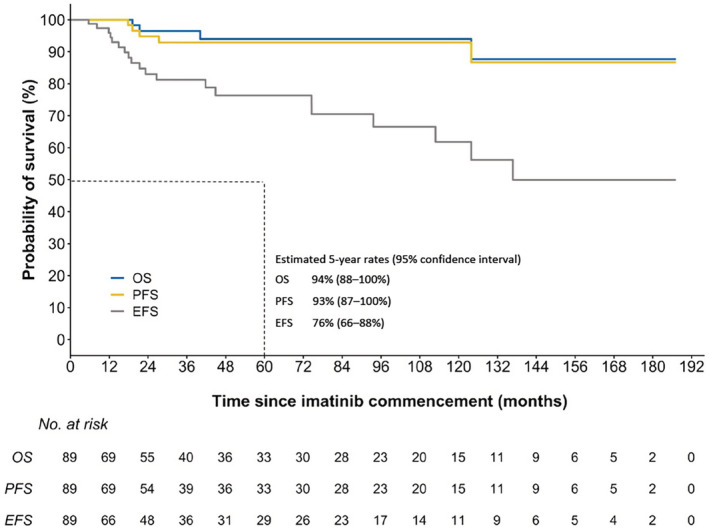
Kaplan–Meier estimated overall survival (OS), progression‐free survival (PFS) and event free survival (EFS) in patients receiving imatinib treatment.

Within the follow‐up period (median 33 months, IQR 14 to 102 months), one patient's disease had progressed from chronic phase to accelerated phase CML, whilst another patient's disease had transformed to AML resulting in death approximately 1 year later. An additional three patients died, with recorded cause(s) of death including cardiac events (*n* = 2) and head injury post fall (*n* = 1), and an additional 15 patients experienced molecular or hematological relapse. Estimated OS, PFS, and EFS rates at 3 years were 97% (95% CI, 92% to 100%), 93% (95% CI, 87% to 100%), and 81% (95% CI, 72% to 92%), respectively (Figure 4). Estimated 5‐year OS, PFS, and EFS rates were 94% (95% CI, 88% to 100%), 93% (95% CI, 87% to 100%), and 76% (95% CI, 66% to 88%), respectively (Figure 4).

A univariable regression analysis identified the following predictors of EFS with imatinib treatment: Sokal score, pre‐existing cardiovascular disease, and imatinib in combination with another antineoplastic agent for CML (Table S5). In the multivariable model, after adjusting for baseline Sokal score and line of treatment, pre‐existing cardiovascular disease was the only independent predictor for inferior EFS with imatinib treatment (HR_adjusted_, 3.18; 95% CI, 1.22 to 8.24; *p* < .05).

### 3.5 Considering clinical trial exclusion criteria

Overall, 48 patients treated with imatinib (56%) would have been excluded from the DASISION and ENESTnd trials due to serious or poorly controlled medical conditions (*n* = 44, 51% of patients), inadequate hepatic or renal function (*n* = 9, 10%), concurrent cancer (*n* = 1, 1%), concomitant use of therapeutic coumarin derivatives (*n* = 7, 8%), or receiving treatment with any medicines that are CYP3A4 inhibitors or inducers (*n* = 7, 8%).

Patients who would have been ineligible for both DASISION and ENESTnd clinical trials (based on the exclusion criteria) were significantly older (mean age 64 vs. 44 years if eligible, *p* < .001), had a higher baseline CCI score (median score 5 vs. 2 if eligible, *p* < .001), and had a higher‐risk according to the ELTS score (Intermediate‐high risk: 60% vs. 37% if eligible; *p* < .05; Table 1). A significantly larger proportion of patients in the ineligible group were receiving one or more potentially interacting medicine during imatinib treatment (87% vs. 51% if eligible; *p* < .001). Other baseline characteristics, including total body weight, imatinib starting dose, distribution of the ECOG PS and Sokal risk scores, were well balanced between groups.

**FIGURE 5:**
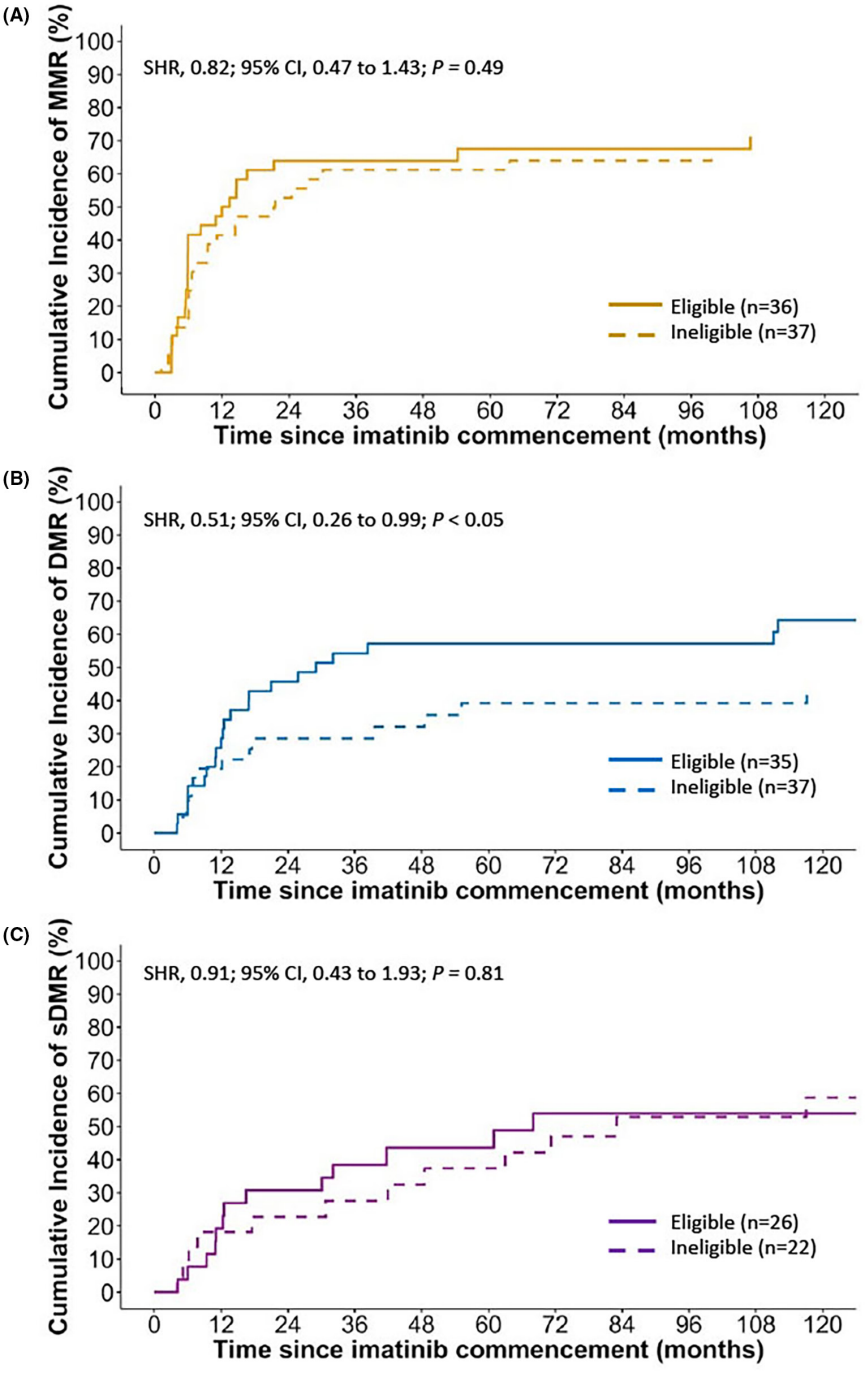
Cumulative incidence of (A) major molecular response (MMR), (B) deep molecular response (DMR) and (C) sustained DMR (sDMR) in patients treated with imatinib, by likely eligibility for the ENESTnd^37^ and DASISION^38^ trials. The unadjusted subdistribution hazard ratios (SHRs) and associated 95% confidence intervals (CIs) are reported, with Gray's weighted log‐rank test used to compare groups.

**FIGURE 6:**
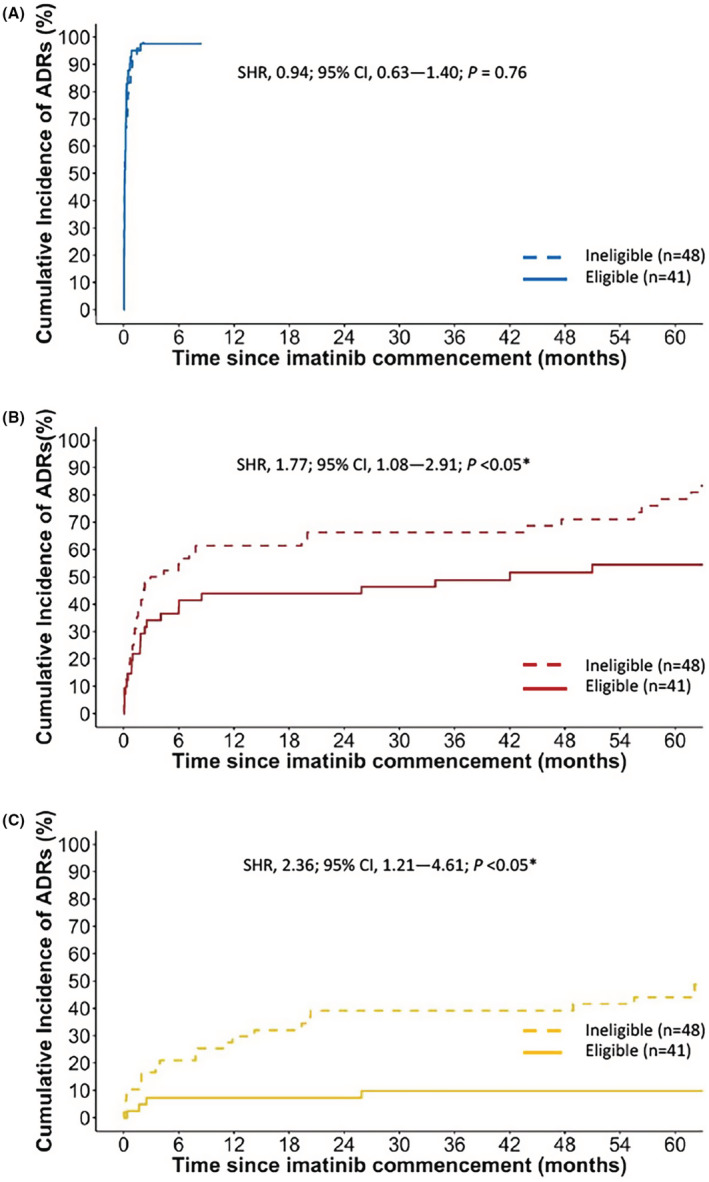
Cumulative incidence of imatinib‐related adverse drug reactions (ADRs) by likely eligibility for the ENESTnd^37^ and DASISION^38^ trials; (A) ADRs of any grade, (B) grade ≥3 ADRs and (C) ADRs resulting in hospitalizationThe unadjusted subdistribution hazard ratios (SHRs) and associated 95% confidence intervals (CIs) are reported, with Gray's weighted log‐rank test used to compare groups.

**FIGURE 7:**
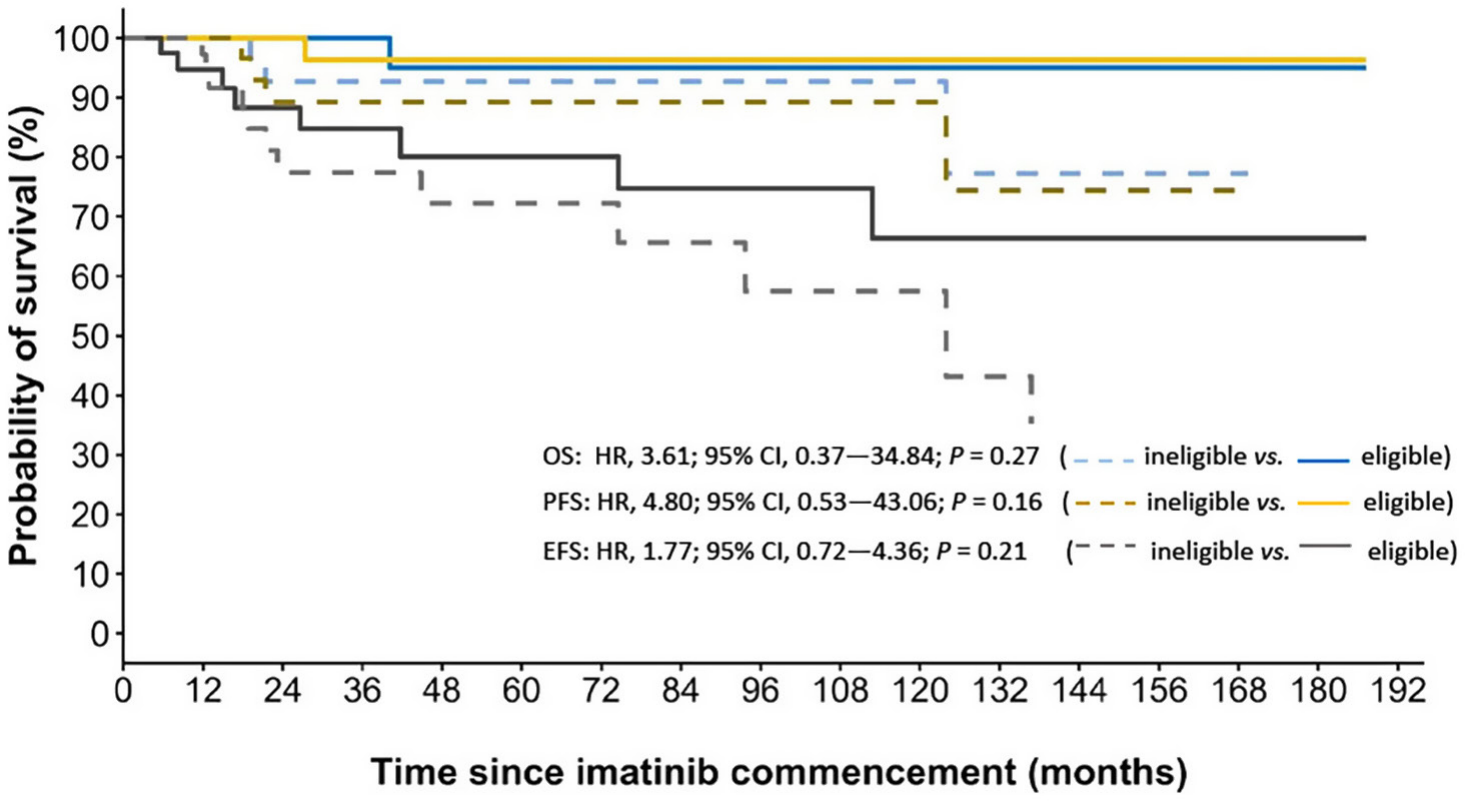
Kaplan–Meier estimated overall survival (OS), progression‐free survival (PFS), and event‐free survival (EFS) in imatinib‐treated patients by likely eligibility for the ENESTnd^37^ and DASISION^38^ trials. The unadjusted hazard ratios (HRs) and associated 95% confidence intervals (CIs) are reported, with a log‐rank test used to compare groups. 48 treatments likely ineligible for the ENESTnd and DASISION trials (dashed lines) and 41 likely eligible (solid lines).

Of interest, patients likely ineligible for the ENESTnd and DASISION trials had a significantly higher risk of recurrent imatinib dose reductions or temporary treatment interruptions (HR, 1.53; 95% CI, 0.80 to 2.90; *p* < .05) compared to patients who would have been eligible. Although MMR rates were comparable between groups (SHR, 0.82; 95% CI, 0.47 to 1.43; *p* = .49; Figure 5), inferior DMR rates at any time were observed in patients considered ineligible for the ENESTnd and DASISION trials (SHR, 0.51; 95% CI, 0.26 to 0.99; *p* < .05; Figure 5). Furthermore, the ineligible cohort had a significantly higher risk of occurrence of imatinib‐related grade ≥ 3 ADRs (SHR, 1.77; 95% CI, 1.08 to 2.91; *p* < .05; Table 2; Figure 6), specifically non‐hematological grade ≥ 3 ADRs (SHR, 2.27; 95% CI, 1.29 to 4.00; *p* < .05), and ADRs resulting in hospitalization (SHR, 2.36; 95% CI, 1.21 to 4.61; *p* < .05; Figure 6). Patients considered ineligible for clinical trial inclusion were also more likely to experience recurrent imatinib‐related ADRs (Table 2), including recurrent grade ≥ 3 ADRs (HR, 1.45; 95% CI, 1.05 to 2.01; *p* < .05) and recurrent ADRs resulting in hospitalization (HR, 1.89; 95% CI, 1.06 to 3.39; *p* < .05). No significant differences were observed in OS, PFS and EFS after eligibility criteria of ENESTnd and DASISION trials were applied to patients in our study (Figure 7).

## 4 DISCUSSION

This study shows a high rate of molecular response and good long‐term survival with imatinib treatment for people with CML in real‐world clinical practice. Survival outcomes with imatinib‐treatment in this study (3‐year OS of 95%, PFS of 93%, and EFS of 81%) are consistent with results of other real‐world^48^ and clinical trial^39^, ^49^, ^50^, ^51^, ^52^ data. In the ENESTnd trial, the estimated 3‐year OS, PFS and EFS rates for patients treated with imatinib were 94%, 94%, and 93% respectively.^49^ For patients receiving imatinib in the DASISION trial, the 3‐year OS and PFS rates were 93% and 91%, respectively.^50^ Similar survival probabilities were noted in the Tyrosine Kinase Inhibitor Optimization and Selectivity (TOPS) trial (3‐year OS, PFS and EFS of 96%, 94% and 92% in the imatinib 400 mg/day arm, respectively, and 96%, 97% and 95% in the imatinib 800 mg/day arm, respectively).^51^ Minor differences in survival outcomes could be explained by differences in definitions and censoring between studies.

Notably, major and deep molecular response rates observed in this study (3‐year MMR and DMR rates of 63% and 42%, respectively) are higher than those previously reported in controlled clinical trials.^49^, ^50^, ^51^, ^53^, ^54^ In the ENESTnd and DASISION trials, 3‐year cumulative MMR rates of imatinib‐treated patients were 53% and 55%, respectively, whilst 3‐year cumulative DMR rates were 26% and 14%, respectively.^49^, ^50^ Similarly, the rate of MMR at 3‐years in the TOPS trial was 52% for the imatinib 400 mg/day arm and 50% for the imatinib 800 mg/day arm, with DMR achieved in 13% of patients in both the 400 and 800 mg/day treatment groups.^51^ Our findings are also consistent with a study of 208 patients treated with first‐line imatinib outside clinical trials which reported estimated 7‐year MMR and DMR rates of 70% and 52%, respectively.^55^

Achievement of sDMR has been associated with significant improvements in long‐term survival outcomes with imatinib,^3^, ^53^, ^56^ and is considered the gateway to obtaining TFR.^57^ Although elective discontinuation of imatinib due to attainment of sDMR was only considered in a small sample of patients in this real‐world cohort, we observed a similar rate of remission after imatinib discontinuation to previous reports (12‐month TFR rate between 41% to 68%).^58^, ^59^, ^60^, ^61^, ^62^, ^63^, ^64^, ^65^ This raises the possibility that, at least in some patients, CML may be cured with imatinib treatment. Imatinib discontinuation has a large economic impact, with cost analysis of the Euro‐SKI (European Stop Tyrosine Kinase Inhibitor) study demonstrating substantial cost savings of €22 million with imatinib discontinuation.^63^ Multiple clinical parameters have been identified as potential predictors of TFR after imatinib discontinuation, including; longer treatment duration, longer DMR durations, previous interferon‐α treatment, low Sokal score, male sex and older age.^58^, ^59^, ^62^, ^63^, ^65^, ^66^ Predictors of TFR were not explored in this study due to the small number of patients attempting discontinuation after sDMR achievement.

The nature of ADRs experienced with imatinib in this study is consistent with observations in controlled clinical trials, with no new safety signals identified. The majority of ADRs occurred within the first 6 months of imatinib therapy and very few new ADRs were reported after 12 months on therapy. However, the 3‐year probability of imatinib‐related grade ≥ 3 ADRs in this study was notably higher than observed with imatinib‐treated patients in ENESTnd and DASISION (58% vs. 24% and 28%, respectively).^49^, ^50^ Specifically, the frequencies of non‐hematological grade ≥ 3 ADRs observed in this real‐world study were higher than that reported in clinical trials. In an 18‐month follow‐up of the IRIS trial, non‐hematological grade ≥ 3 ADRs had occurred in 14% of imatinib‐treated patients (vs. 38% by 18 months in this study), with grade ≥ 3 rash in 2% (vs. 13% by 18 months) and superficial oedema in 1% (vs. 5% by 18 months).^1^ Imatinib‐treated patients in DASISION^38^ and ENESTnd^37^ had similarly low incidences of non‐hematological grade ≥ 3 ADRs, with grade ≥ 3 rash occurring in 1% and superficial oedema in <1% of patients within a median follow‐up of 14 months. The incidence of hematological grade ≥ 3 ADRs in this study reflects that reported in clinical trials.^1^, ^37^, ^38^

A major finding of this study is the high incidence of ADRs resulting in imatinib discontinuation, with an 18‐month probability of 15% and 3‐year probability of 25%. This is notably higher than previous reports. In the 3‐year follow‐up of the ENESTnd and DASISION trials,^49^, ^50^ 11% and 6% of imatinib treated patients, respectively, had discontinued treatment due to ADRs. Similarly, low rates of imatinib discontinuation secondary to ADRs were reported in the TOPS trial (4% of imatinib 400 mg/day and 9% of imatinib 800 mg/day, 17 month median follow‐up).^67^ In a phase 2 study of imatinib treatment post interferon‐ α failure, ADRs let to imatinib discontinuation in only 2% of patients (median follow‐up of 18 months).^68^ Analysis of the French subset of the UNIC (Unmet needs in CML) study reported that 31% of patients receiving imatinib had experienced an ADR leading to dose changes or discontinuation of imatinib within a median treatment duration of 3 years,^69^ which is notably lower than the 3‐year rate of 58% observed in this study.

This study also identified a range of implications of imatinib‐related ADRs on healthcare resource utilization in real‐world patients treated in an Australian setting. The French subset of the UNIC study also reported high healthcare utilization in patients receiving imatinib, with a mean number of 3 general practitioner visits, 5 hematology visits, 1 visit to another specialist, 1 outpatient hospital stay, 0.5 chest x‐rays, 0.2 blood transfusions, and 0.2 computerized tomography scans required during the last 12 months of each patients' observation period.^69^

There is large inter‐individual variability in imatinib PK, with reported coefficient of variations of 40 to 60% observed in imatinib trough plasma concentrations at steady state (C_ss,min_) in patients administered the same dose.^70^, ^71^, ^72^, ^73^, ^74^ Variability in imatinib PK is a possible determinant of variability in imatinib efficacy and toxicity.^70^, ^71^, ^73^, ^75^, ^76^, ^77^, ^78^, ^79^, ^80^, ^81^, ^82^ Although plasma concentration data were not available for this cohort, we conducted a follow‐up study using physiologically based pharmacokinetic (PBPK) modeling and simulation to predict the imatinib plasma concentration‐time profile of patients included in this real‐world study.^83^ Notably, the PBPK model showed significant correlations between predicted steady‐state imatinib exposure and clinical outcomes (achievement of EMR, and the occurrence of grade ≥ 3 imatinib‐related ADRs).^83^

In multivariable regression analyses, lower total body weight was predictive of higher rates of occurrence of imatinib‐related grade ≥ 3 ADRs. This is supported in a study by Shin et al^84^ where patients with a lower total body weight (≤ 64 kg) had a significantly higher incidence of imatinib‐related dose‐limiting toxicities compared to heavier patients (58% vs. 30% at 12 months, *p* < .001). This effect could be a result of higher imatinib plasma concentrations, and therefore more frequent toxicities, in patients with lower total body weight.^70^, ^75^, ^76^, ^82^ Female sex was independently predictive of recurrent imatinib‐related grade ≥ 3 ADRs, consistent with previous observations.^39^, ^85^ Of importance, females in this study had significantly lower total body weight compared to males (median 65 vs. 82 kg, *p* < .001), and thus possibly higher systemic exposure. Imatinib plasma concentrations up to 30% higher have been reported in females compared with males.^76^, ^86^, ^87^

This study adds to the evidence that geographic ancestry is an important covariate in the inter‐individual variability of imatinib treatment outcomes.^88^ A significantly higher hazard of imatinib‐related grade ≥ 3 ADRs was observed in patients of Middle Eastern/North African or Pacific Islander ancestries, compared to patients of European ancestry. This is a novel finding, however with small numbers of patients in these groups, further studies with a larger sample size are required to confirm this observation. Furthermore, East Asian patients had a trend towards higher likelihood of recurrent imatinib‐related grade ≥ 3 ADRs compared to patients of European ancestry. Similarly, subpopulation analyses of the DASISION and ENESTnd trials revealed that patients of East Asian ancestry were more susceptible than non‐East Asian patients to imatinib‐related fluid retention, rash, nausea, and grade ≥ 3 neutropenia, and more frequently required an imatinib dose reduction or temporary treatment interruption.^89^, ^90^, ^91^ This variability in response is possibly a reflection of inter‐ethnic differences in PK determinants of imatinib, such as in the expression/activity of breast cancer resistance protein (BCRP) and P‐glycoprotein (P‐gp), plasma protein binding, body size and weight, complementary medicine use, and diet.^88^ Despite these observations, there are currently no guidelines recommending dose adjustment of imatinib in different ethnic groups. Body‐weight based dosing, combined with therapeutic drug monitoring‐guided dose adaptation, may help reduce the incidence of severe imatinib‐related ADRs in patients of East‐Asian ancestry.

Comorbidities are important considerations in treatment of diseases with onset in older populations and expected long survival times, such as CML. An important finding of this study is the strong association between comorbidities at diagnosis and inferior efficacy and tolerability with imatinib treatment. Similarly, in the German CML‐Study IV, a higher CCI score was associated with lower OS probabilities in multivariable analysis, even after removal of age‐related components from the score.^92^ A retrospective study in Brazil found significantly poorer EFS with imatinib and a higher rate of temporary treatment interruption secondary to toxicities and nonadherence in patients with higher CCI scores.^93^ CCI stratification applied on a large cohort of older patients with CML (> 75 years) receiving imatinib treatment reported a significant correlation between CCI and survival (EFS and OS).^94^ We hypothesize that the increased risk of recurrent imatinib‐related grade ≥ 3 ADRs observed in patients with higher CCI scores and pre‐existing pulmonary disease resulted in poor adherence or more frequent imatinib dose reductions/interruptions, leading to inferior molecular response rates and EFS.

Interestingly, receiving a concomitant potentially interacting medicine during imatinib treatment (71% of patients) was predictive of superior MMR rates and occurrence of imatinib‐related grade ≥ 3 ADRs in this real‐world cohort. Few studies have explored the effect of concomitant medicines on imatinib outcomes, with none selecting for medicines with the potential for PK or PD drug–drug interactions.^95^, ^96^ Kunbaz et al^96^ showed that receiving ≥1 concomitant medicines for management of comorbidities was associated with a higher incidence of imatinib‐related ADRs, whilst Gora‐Tybor et al^95^ showed a higher probability of molecular response in patients receiving ≥2 concomitant medicines with imatinib treatment. Imatinib is predominantly metabolized by CYP3A4 and CYP2C8 to a major metabolite N‐desmethyl imatinib, and is a substrate of P‐gp and BCRP efflux transporters.^97^ Coadministering inhibitors or inducers of these enzymes and transporters with imatinib has the potential to alter imatinib systemic exposure, which is particularly important as imatinib plasma concentrations have been correlated to response rates in CML^70^, ^71^, ^73^, ^76^, ^77^, ^78^, ^79^, ^80^, ^81^ and to ADRs.^70^, ^75^, ^76^, ^82^ Case reports have observed inadequate imatinib response and subtherapeutic imatinib concentrations at standard dosing in patients also receiving treatment with phenytoin^98^ and carbamazepine (CYP3A4 inducers).^99^, ^100^, ^101^ Additionally, as a mechanism based inhibitor of CYP3A4,^102^ imatinib itself can also influence the systemic exposure of concomitantly administered CYP3A4 substrates,^103^, ^104^ potentially altering their toxicity and response. A case report of imatinib coadministered with cyclosporine, a CYP3A4 substrate, reported an increased plasma concentration of cyclosporine and increased need for cyclosporine dose reduction due to toxicities.^105^ Therefore, drug–drug interactions should be considered as a potential source of toxicities or inadequate response with imatinib treatment and consideration of alternatives to avoid interactions is recommended.

The finding of superior imatinib response with a low‐risk ELTS score is consistent with recent reports in real‐world settings^106^, ^107^, ^108^, ^109^ and clinical‐trial populations.^21^ Baseline *BCR‐ABL1* transcript type is another CML disease prognostic marker. Contrary to results from this study, superior MMR rates with imatinib treatment have been demonstrated in patients expressing the e14a2 transcript compared to e13a2 transcript.^53^, ^110^, ^111^, ^112^, ^113^, ^114^, ^115^, ^116^ The negative correlation found in this study between MMR and e14a2 *BCR‐ABL1* transcript type could possibly reflect differences in other baseline characteristics. For example, patients expressing the e14a2 transcript were younger than e13a2 expressors (median age 56 vs. 63 years), with a higher ECOG PS (median score of 1 vs. 0) and a lower proportion of potential imatinib‐drug interactions (61 vs 78%).

Results from this study indicate that the approach of using a standard 400 mg/day imatinib starting dose for patients with chronic phase CML can be improved. Higher imatinib starting doses of 600 or 800 mg/day were associated with significantly superior EMR and MMR rates compared to doses of 400 or 500 mg/day, in agreement with other studies. Single‐arm observational studies of patients receiving high‐dose imatinib (800 mg/day) have found higher rates of MMR and CCyR when compared with historical controls or patients from the IRIS study treated with 400 mg daily.^117^, ^118^, ^119^ A single institution study observed superior CCyR rates in patients receiving an average imatinib dose greater than 350 mg/day during the first 6 months of first‐line treatment.^54^ Imatinib dose escalation to 600 mg/day in patients failing to achieve optimal response with 400 mg/day has also proven to significantly increase MMR and CCyR rates.^120^, ^121^ Furthermore, randomized controlled trials comparing imatinib starting doses of 800 mg/day with standard doses of 400 mg/day have demonstrated quicker and deeper MMR and CCyR with high‐dose imatinib.^53^, ^56^, ^67^, ^122^ However, improved efficacy is likely to come at the cost of tolerability. In concordance with previous findings,^67^, ^122^ in our study higher imatinib starting doses (600 or 800 mg/day) were predictive of grade ≥ 3 ADRs in this real‐world cohort. A refinement of clinical practice that may minimize toxicity could be to initiate imatinib at a high‐dose (800 mg/day) and aim to dose reduce after achievement of a stable molecular response, with studies demonstrating maintenance of molecular response in the majority of patients reduced to imatinib 300 or 400 mg/day.^123^, ^124^

It is widely acknowledged that the study population in clinical trials does not reflect the community of patients requiring treatment. In our study, we demonstrated that over half of real‐world treated patients on imatinib would have been excluded from participation in the seminal phase III ENESTnd and DASISION studies. Those patients likely to be excluded were older, had a higher comorbidity burden, higher ELTS risk score, and a larger proportion received concomitant potentially interacting medicines. Accordingly, compared with the real‐world patients who would have been eligible for the clinical trials, these patients demonstrated inferior DMR rates and a higher risk of grade ≥ 3 ADRs. Rates of MMR were similar between groups, as was duration of PFS, whilst the availability of potent second‐line TKIs is a likely explanation for preserved OS in this group. Another retrospective observational study reported that 14% of patients would have been excluded from both the ENESTnd and DASISION trials due to severe comorbidities.^125^ Patients who would have been excluded were significantly older, with a higher CCI score, higher Sokal risk, higher number of concomitant medicines, and showed inferior treatment outcomes (a higher rate of severe ADRs and worse OS).^125^ The applicability of all results from controlled clinical trials to complex patients in real‐world clinical practice, with many competing risks, should therefore be exercised with caution. Our study confirms the utility of real‐world post‐marketing studies which include subsets of patients often excluded from trials.

As CML is considered a rare disease, the sample size of this study was limited by the number of patients diagnosed and treated at the respective centres over the period of data collection. As such, the effect of certain baseline predictor variables (e.g. disease phase, BM fibrosis, additional BM karyotype abnormalities, body mass index, body surface area) on imatinib clinical outcomes could not be evaluated in multivariable regression. As a retrospective study, the information available relies on the reporting by patients and physicians at the time, leading to a potential underreporting of imatinib‐related ADRs. Furthermore, patient adherence to their prescribed imatinib dose regimens could not be considered in regression models of imatinib treatment outcomes due to lack of sufficient recording in medical records. Owing to their therapeutic success, only a small number of patients died and/or experienced disease progression on imatinib treatment. As such, large patient samples and long observation times are needed to identify groups with different OS and PFS. Finally, outcomes from this observational study cannot be compared directly with those from other studies without acknowledging differences in study design (including lack of randomization in this real‐world study, and differential monitoring of outcomes and management of events) and definitions of outcome measures.

Despite these limitations, the similarity in survival results and adverse event profiles with those of controlled clinical trials provides a level of confidence in the data, with differences observed likely to reflect true differences between real‐world and protocol‐driven practices. Importantly, characteristics of the patients included in this study were consistent with expectations of a CML patient receiving care in the Australian oncology setting.^126^ Conversely, patients included in the IRIS, ENESTnd, and DASISION controlled trials were younger than expected in a real‐world setting (median age of 50 years in IRIS,^1^ 46 years in ENESTnd,^37^ and 49 years in DASISION).^38^ There are several strengths in the methodology of this study, including use of the CTCAE and Naranjo algorithm to classify ADRs, re‐abstraction of data by a second investigator in 30% of randomly selected patients, use of the cumulative incidence competing risk method to evaluate molecular response rates and ADR incidences, and use of a multiple imputation method in cases of missing data. Importantly, this real‐world data on 89 imatinib treatment courses represents a total of 421 patient years of experience with imatinib treatment in CML. As a real‐world study, this data has presented new and important insights into prescribing practices and clinical outcomes of patients receiving imatinib treatment with complex comorbidities and on multiple medicines, without the potential selection bias present in controlled clinical trials.

In summary, this study found that imatinib induces fast and deep molecular responses that translate to good survival outcomes in a real‐world setting. A higher incidence of imatinib‐related ADRs were observed in this real‐world cohort, compared to controlled clinical trials. Baseline evaluation of concomitant medicine use and pre‐existing comorbidities, together with consideration of biological and clinical factors, can help identify patients with an excellent prognosis and those who may require careful monitoring and/or intervention. Early high‐doses of imatinib, followed by rapid individualized dose‐adaptation to good tolerability can be a strategy to achieve a balance between efficacy and tolerability.

## AUTHOR CONTRIBUTIONS

All authors conceived the study. J.A collected, analyzed, and interpreted the data, and wrote the manuscript. A.G, A.M, and N.W.D also contributed to the interpretation of the data and revised the manuscript. All authors approved the final manuscript.

## FUNDING INFORMATION

This work was supported by the Peter Coates Postgraduate Scholarship in Ethnopharmacology provided by GlaxoSmithKline.

## DISCLOSURE

The authors declare that the research was conducted in the absence of any commercial or financial relationships that could be construed as a potential conflict of interest.

## ETHICS APPROVAL AND PATIENT CONSENT STATEMENT

The research included in this study was approved by the Sydney Local Health District Human Research Ethics Committee (reference number: LNR/17/CRGH/248). Site specific approval was also obtained for Concord Repatriation General Hospital (reference number: LNRSSA/17/CRGH/249) and Royal North Shore Hospital (reference number: RESP/18/146). A waiver of consent according to the National Statement on Ethical Conduct in Human Research was granted by the Human Research Ethics Committee.

## Supporting information


Appendix S1

## Data Availability

The data that support the findings of this study are available on request from the corresponding author. The data are not publicly available because of privacy or ethical restrictions.

## References

[1] O'Brien SG , Guilhot F , Larson RA , et al. Imatinib compared with interferon and low‐dose cytarabine for newly diagnosed chronic‐phase chronic myeloid leukemia. N Engl J Med. 2003;348(11):994‐1004. doi:10.1056/NEJMoa022457 12637609

[2] Vener C , Banzi R , Ambrogi F , et al. First‐line imatinib vs second‐ and third‐generation TKIs for chronic‐phase CML: a systematic review and meta‐analysis. Blood Adv. 2020;4(12):2723‐2735. doi:10.1182/bloodadvances.2019001329 32559295 PMC7322957

[3] Druker BJ , Guilhot F , O'Brien SG , et al. Five‐year follow‐up of patients receiving imatinib for chronic myeloid leukemia. N Engl J Med. 2006;355(23):2408‐2417. doi:10.1056/NEJMoa062867 17151364

[4] Deininger M , O'Brien SG , Guilhot F , et al. International randomized study of interferon vs STI571 (IRIS) 8‐year follow up: sustained survival and low risk for progression or events in patients with newly diagnosed chronic myeloid leukemia in chronic phase (CML‐CP) treated with imatinib. Blood. 2009;114(22):1126. doi:10.1182/blood.V114.22.1126.1126

[5] Branford S , Yeung DT , Ross DM , et al. Early molecular response and female sex strongly predict stable undetectable BCR‐ABL1, the criteria for imatinib discontinuation in patients with CML. Blood. 2013;121(19):3818‐3824. doi:10.1182/blood-2012-10-462291 23515925

[6] Hochhaus A , Breccia M , Saglio G , et al. Expert opinion—management of chronic myeloid leukemia after resistance to second‐generation tyrosine kinase inhibitors. Leukemia. 2020;34(6):1495‐1502. doi:10.1038/s41375-020-0842-9 32366938 PMC7266739

[7] Cancer Institute NSW . Enrolments in Cancer Clinical Trials as a Proportion of Cancer Incidence. NSW Government. Accessed November 9, 2020. https://www.cancer.nsw.gov.au/what‐we‐do/nsw‐cancer‐plan/performance‐index/enrolments‐in‐cancer‐clinical‐trials‐as‐a‐proporti

[8] U.S. Food and Drug Administration . Framework for FDA's Real‐World Evidence Program. Food and Drug Administration; 2018. Updated December 01, 2018. Accessed November 10, 2020. https://www.fda.gov/media/120060/download

[9] Blonde L , Khunti K , Harris SB , Meizinger C , Skolnik NS . Interpretation and impact of real‐world clinical data for the practicing clinician. Adv Ther. 2018;35(11):1763‐1774. doi:10.1007/s12325-018-0805-y 30357570 PMC6223979

[10] De Rawlins M . Testimonio: on the evidence for decisions about the use of therapeutic interventions. Clin Med (Lond). 2008;8(6):579‐588. doi:10.7861/clinmedicine.8-6-579 19149278 PMC4954394

[11] Jin S , Pazdur R , Sridhara R . Re‐evaluating eligibility criteria for oncology clinical trials: analysis of investigational new drug applications in 2015. J Clin Oncol. 2017;35(33):3745‐3752. doi:10.1200/JCO.2017.73.4186 28968168 PMC5692723

[12] Ludmir EB , Mainwaring W , Lin TA , et al. Factors associated with age disparities among cancer clinical trial participants. JAMA Oncol. 2019;5(12):1769‐1763. doi:10.1001/jamaoncol.2019.2055 31158272 PMC6547133

[13] Nanchahal K , Mangtani P , Alston M , dos Santos SI . Development and validation of a computerized south Asian names and group recognition algorithm (SANGRA) for use in British health‐related studies. J Public Health Med. 2001;23(4):278‐285. 11873889 10.1093/pubmed/23.4.278

[14] Macfarlane GJ , Lunt M , Palmer B , Afzal C , Silman AJ , Esmail A . Determining aspects of ethnicity amongst persons of south Asian origin: the use of a surname‐classification programme (Nam Pehchan). Public Health. 2007;121(3):231‐236. doi:10.1016/j.puhe.2006.07.001 17240412

[15] Rezai MR , Maclagan LC , Donovan LR , Tu JV . Classification of Canadian immigrants into visible minority groups using country of birth and mother tongue. Open Med. 2013;7(4):e85‐e93. 25237404 PMC4161499

[16] Charlson ME , Pompei P , Ales KL , MacKenzie CR . A new method of classifying prognostic comorbidity in longitudinal studies: development and validation. J Chronic Dis. 1987;40(5):373‐383. doi:10.1016/0021-9681(87)90171-8 3558716

[17] Oken MM , Creech RH , Tormey DC , et al. Toxicity and response criteria of the eastern cooperative oncology group. Am J Clin Oncol. 1982;5(6):649‐655. 7165009

[18] Deininger MW , Shah NP , Altman JK , et al. Chronic myeloid leukemia, version 2.2021, NCCN clinical practice guidelines in oncology. J Natl Compr Canc Netw. 2020;18(10):1385‐1415. doi:10.6004/jnccn.2020.0047 33022644

[19] Baccarani M , Pileri S , Steegmann JL , Muller M , Soverini S , Dreyling M . Chronic myeloid leukemia: ESMO clinical practice guidelines for diagnosis, treatment and follow‐up. Ann Oncol 2012;23 (suppl 7):72–77. Accessed October 10, 2020. https://www.annalsofoncology.org/article/S0923‐7534(19)37658‐6/fulltext 22997458 10.1093/annonc/mds228

[20] Sokal JE , Cox EB , Baccarani M , et al. Prognostic discrimination in "good‐risk" chronic granulocytic leukemia. Blood. 1984;63(4):789‐799. 6584184

[21] Pfirrmann M , Baccarani M , Saussele S , et al. Prognosis of long‐term survival considering disease‐specific death in patients with chronic myeloid leukemia. Leukemia. 2016;30(1):48‐56. doi:10.1038/leu.2015.261 26416462

[22] Naranjo CA , Busto U , Sellers EM , et al. A method for estimating the probability of adverse drug reactions. Clin Pharmacol Ther. 1981;30(2):239‐245. doi:10.1038/clpt.1981.154 7249508

[23] World Health Organization . Requirements for Adverse Drug Reaction Reporting. World Health Organization; 1975.

[24] National Cancer Institute (U.S.) . Common Terminology Criteria for Adverse Events (CTCAE) v5.0. U.S. Department of Health and Human Services; 2017. https://ctep.cancer.gov/protocoldevelopment/electronic_applications/ctc.htm

[25] Kaplan EL , Meier P . Nonparametric estimation from incomplete observations. J Am Stat Assoc. 1958;53(282):457‐481. doi:10.1080/01621459.1958.10501452

[26] Cox DR . Regression models and life‐tables. J R Stat Soc Series B Stat Methodol. 1972;34(2):187‐202. doi:10.1111/j.2517-6161.1972.tb00899.x

[27] Nick TG , Campbell KM . Logistic regression. In: Ambrosius WT , ed. Topics in Biostatistics Methods in Molecular Biology. Humana Press; 2017. doi:10.1007/978-1-59745-530-5_14

[28] Noordzij M , Leffondre K , van Stralen KJ , Zoccali C , Dekker FW , Jager KJ . When do we need competing risks methods for survival analysis in nephrology? Nephrol Dial Transplant. 2013;28(11):2670‐2677. doi:10.1093/ndt/gft355 23975843

[29] Austin PC , Lee DS , Fine JP . Introduction to the analysis of survival data in the presence of competing risks. Circulation. 2016;133(6):601‐609. doi:10.1161/circulationaha.115.017719 26858290 PMC4741409

[30] Schmoor C , Bender R , Beyersmann J , Kieser M , Schumacher M . Adverse event development in clinical oncology trials. Lancet Oncol. 2016;17(7):e263‐e264. doi:10.1016/s1470-2045(16)30223-6 27396638

[31] Fine JP , Gray RJ . A proportional hazards model for the subdistribution of a competing risk. J Am Stat Assoc. 1999;94(446):496‐509. doi:10.1080/01621459.1999.10474144

[32] Scrucca L , Santucci A , Aversa F . Regression modeling of competing risk using R: an in depth guide for clinicians. Bone Marrow Transplant. 2010;45(9):1388‐1395. doi:10.1038/bmt.2009.359 20062101

[33] Scrucca L , Santucci A , Aversa F . Competing risk analysis using R: an easy guide for clinicians. Bone Marrow Transplant. 2007;40(4):381‐387. doi:10.1038/sj.bmt.1705727 17563735

[34] Zhang X , Zhang MJ , Fine J . A proportional hazards regression model for the subdistribution with right‐censored and left‐truncated competing risks data. Stat Med. 2011;30(16):1933‐1951. doi:10.1002/sim.4264 21557288 PMC3408877

[35] Allignol A , Beyersmann J , Schmoor C . Statistical issues in the analysis of adverse events in time‐to‐event data. Pharm Stat. 2016;15(4):297‐305. doi:10.1002/pst.1739 26929180

[36] Prentice RL , Williams BJ , Peterson AV . On the regression analysis of multivariate failure time data. Biometrika. 1981;68(2):373‐379. doi:10.2307/2335582

[37] Saglio G , Kim DW , Issaragrisil S , et al. Nilotinib versus imatinib for newly diagnosed chronic myeloid leukemia. N Engl J Med. 2010;362(24):2251‐2259. doi:10.1056/NEJMoa0912614 20525993

[38] Kantarjian H , Shah NP , Hochhaus A , et al. Dasatinib versus imatinib in newly diagnosed chronic‐phase chronic myeloid leukemia. N Engl J Med. 2010;362(24):2260‐2270. doi:10.1056/NEJMoa1002315 20525995

[39] Kalmanti L , Saussele S , Lauseker M , et al. Safety and efficacy of imatinib in CML over a period of 10 years: data from the randomized CML‐study IV. Leukemia. 2015;29(5):1123‐1132. doi:10.1038/leu.2015.36 25676422

[40] Hochhaus A , Baccarani M , Silver RT , et al. European LeukemiaNet 2020 recommendations for treating chronic myeloid leukemia. Leukemia. 2020;34(4):966‐984. doi:10.1038/s41375-020-0776-2 32127639 PMC7214240

[41] Haouala A , Widmer N , Duchosal MA , Montemurro M , Buclin T , Decosterd LA . Drug interactions with the tyrosine kinase inhibitors imatinib, dasatinib, and nilotinib. Blood. 2011;117(8):e75‐e87. doi:10.1182/blood-2010-07-294330 20810928

[42] van Buuren S , Groothuis‐Oudshoorn K . Mice: multivariate imputation by chained equations in R. J Stat Soft. 2011;45(3):67. doi:10.18637/jss.v045.i03

[43] Greenland S , Finkle WD . A critical look at methods for handling missing covariates in epidemiologic regression analyses. Am J Epidemiol. 1995;142(12):1255‐1264. doi:10.1093/oxfordjournals.aje.a117592 7503045

[44] Beesley LJ , Bartlett JW , Wolf GT , Taylor JM . Multiple imputation of missing covariates for the Cox proportional hazards cure model. Stat Med. 2016;35(26):4701‐4717. doi:10.1002/sim.7048 27439726 PMC5053880

[45] R Core Team . R: A Language and Environment for Statistical Computing. R Foundation for Statistical Computing; 2020. http://www.R‐project.org/

[46] Harding SD , Sharman JL , Faccenda E , et al. The IUPHAR/BPS guide to PHARMACOLOGY in 2019: updates and expansion to encompass the new guide to IMMUNOPHARMACOLOGY. Nucleic Acids Res. 2018;46:D1091‐D1106. doi:10.1093/nar/gkx1121 29149325 PMC5753190

[47] Alexander SPH , Kelly E , Mathie A , et al. THE CONCISE GUIDE TO PHARMACOLOGY 2021/22: introduction and other protein targets. Br J Pharmacol. 2021;178:S1‐S26. doi:10.1111/bph.15537 34529830 PMC9513948

[48] Geelen IG , Thielen N , Janssen JJ , et al. Treatment outcome in a population‐based, ‘real‐world’ cohort of patients with chronic myeloid leukemia. Haematologica. 2017;102(11):1842‐1849. doi:10.3324/haematol.2017.174953 28860339 PMC5664388

[49] Larson RA , Hochhaus A , Hughes TP , et al. Nilotinib vs imatinib in patients with newly diagnosed Philadelphia chromosome‐positive chronic myeloid leukemia in chronic phase: ENESTnd 3‐year follow‐up. Leukemia. 2012;26(10):2197‐2203. doi:10.1038/leu.2012.134 22699418

[50] Jabbour E , Kantarjian HM , Saglio G , et al. Early response with dasatinib or imatinib in chronic myeloid leukemia: 3‐year follow‐up from a randomized phase 3 trial (DASISION). Blood. 2014;123(4):494‐500. doi:10.1182/blood-2013-06-511592 24311723 PMC4190618

[51] Novartis Pharmaceuticals Corporation . Efficacy of 400 mg versus 800 mg imatinib in chronic myeloid leukemia in chronic phase patients ‐ TOPS (Tyrosine Kinase Inhibitor Optimization and Selectivity). Accessed September 10, 2020. https://clinicaltrials.gov/ct2/show/study/NCT00124748

[52] Hochhaus A , Larson RA , Guilhot F , et al. Long‐term outcomes of imatinib treatment for chronic myeloid leukemia. N Engl J Med. 2017;376(10):917‐927. doi:10.1056/NEJMoa1609324 28273028 PMC5901965

[53] Hehlmann R , Müller MC , Lauseker M , et al. Deep molecular response is reached by the majority of patients treated with imatinib, predicts survival, and is achieved more quickly by optimized high‐dose imatinib: results from the randomized CML‐study IV. J Clin Oncol. 2014;32(5):415‐423. doi:10.1200/jco.2013.49.9020 24297946

[54] de Lavallade H , Apperley JF , Khorashad JS , et al. Imatinib for newly diagnosed patients with chronic myeloid leukemia: incidence of sustained responses in an intention‐to‐treat analysis. J Clin Oncol. 2008;26(20):3358‐3363. doi:10.1200/jco.2007.15.8154 18519952

[55] Breccia M , Molica M , Colafigli G , et al. Prognostic factors associated with a stable MR4.5 achievement in chronic myeloid leukemia patients treated with imatinib. Oncotarget. 2017;9(7):7534‐7540. doi:10.18632/oncotarget.23691 29484130 PMC5800922

[56] Hehlmann R , Lauseker M , Jung‐Munkwitz S , et al. Tolerability‐adapted imatinib 800 mg/d versus 400 mg/d versus 400 mg/d plus interferon‐α in newly diagnosed chronic myeloid leukemia. J Clin Oncol. 2011;29(12):1634‐1642. doi:10.1200/jco.2010.32.0598 21422420

[57] Cortes J , Rea D , Lipton JH . Treatment‐free remission with first‐ and second‐generation tyrosine kinase inhibitors. Am J Hematol. 2019;94(3):346‐357. doi:10.1002/ajh.25342 30394563 PMC6587857

[58] Mahon FX , Réa D , Guilhot J , et al. Discontinuation of imatinib in patients with chronic myeloid leukaemia who have maintained complete molecular remission for at least 2 years: the prospective, multicentre stop imatinib (STIM) trial. Lancet Oncol. 2010;11(11):1029‐1035. doi:10.1016/s1470-2045(10)70233-3 20965785

[59] Mori S , Vagge E , le Coutre P , et al. Age and dPCR can predict relapse in CML patients who discontinued imatinib: the ISAV study. Am J Hematol. 2015;90(10):910‐914. doi:10.1002/ajh.24120 26178642

[60] Thielen N , van der Holt B , Cornelissen JJ , et al. Imatinib discontinuation in chronic phase myeloid leukaemia patients in sustained complete molecular response: a randomised trial of the Dutch‐Belgian cooperative trial for Haemato‐oncology (HOVON). Eur J Cancer. 2013;49(15):3242‐3246. doi:10.1016/j.ejca.2013.06.018 23876833

[61] Fava C , Rege‐Cambrin G , Dogliotti I , et al. Observational study of chronic myeloid leukemia Italian patients who discontinued tyrosine kinase inhibitors in clinical practice. Haematologica. 2019;104(8):1589‐1596. doi:10.3324/haematol.2018.205054 30819917 PMC6669161

[62] Etienne G , Guilhot J , Rea D , et al. Long‐term follow‐up of the French stop imatinib (STIM1) study in patients with chronic myeloid leukemia. J Clin Oncol. 2017;35(3):298‐305. doi:10.1200/jco.2016.68.2914 28095277

[63] Saussele S , Richter J , Guilhot J , et al. Discontinuation of tyrosine kinase inhibitor therapy in chronic myeloid leukaemia (EURO‐SKI): a prespecified interim analysis of a prospective, multicentre, non‐randomised, trial. Lancet Oncol. 2018;19(6):747‐757. doi:10.1016/S1470-2045(18)30192-X 29735299

[64] Rousselot P , Charbonnier A , Cony‐Makhoul P , et al. Loss of major molecular response as a trigger for restarting tyrosine kinase inhibitor therapy in patients with chronic‐phase chronic myelogenous leukemia who have stopped imatinib after durable undetectable disease. J Clin Oncol. 2014;32(5):424‐430. doi:10.1200/jco.2012.48.5797 24323036

[65] Ross DM , Branford S , Seymour JF , et al. Safety and efficacy of imatinib cessation for CML patients with stable undetectable minimal residual disease: results from the TWISTER study. Blood. 2013;122(4):515‐522. doi:10.1182/blood-2013-02-483750 23704092

[66] Rinaldetti S , Pfirrmann M , Manz K , et al. Effect of ABCG2, OCT1, and ABCB1 (MDR1) gene expression on treatment‐free remission in a EURO‐SKI subtrial. Clin Lymphoma Myeloma Leuk. 2018;18(4):266‐271. doi:10.1016/j.clml.2018.02.004 29510895

[67] Cortes JE , Baccarani M , Guilhot F , et al. Phase III, randomized, open‐label study of daily imatinib mesylate 400 mg versus 800 mg in patients with newly diagnosed, previously untreated chronic myeloid leukemia in chronic phase using molecular end points: tyrosine kinase inhibitor optimization and selectivity study. J Clin Oncol. 2010;28(3):424‐430. doi:10.1200/JCO.2009.25.3724 20008622 PMC4979244

[68] Kantarjian H , Sawyers C , Hochhaus A , et al. Hematologic and cytogenetic responses to imatinib mesylate in chronic myelogenous leukemia. N Engl J Med. 2002;346(9):645‐652. doi:10.1056/NEJMoa011573 11870241

[69] Michallet M , Tulliez M , Corm S , et al. Management of chronic myeloid leukaemia in clinical practice in France: results of the French subset of patients from the UNIC study. Curr Med Res Opin. 2010;26(2):307‐317. doi:10.1185/03007990903479299 19961284

[70] Larson RA , Druker BJ , Guilhot F , et al. Imatinib pharmacokinetics and its correlation with response and safety in chronic‐phase chronic myeloid leukemia: a subanalysis of the IRIS study. Blood. 2008;111(8):4022‐4028. doi:10.1182/blood-2007-10-116475 18256322

[71] Picard S , Titier K , Etienne G , et al. Trough imatinib plasma levels are associated with both cytogenetic and molecular responses to standard‐dose imatinib in chronic myeloid leukemia. Blood. 2007;109(8):3496‐3499. doi:10.1182/blood-2006-07-036012 17192396

[72] Lankheet NA , Knapen LM , Schellens JH , Beijnen JH , Steeghs N , Huitema AD . Plasma concentrations of tyrosine kinase inhibitors imatinib, erlotinib, and sunitinib in routine clinical outpatient cancer care. Ther Drug Monit. 2014;36(3):326‐334. 24305627 10.1097/FTD.0000000000000004

[73] Bouchet S , Titier K , Moore N , et al. Therapeutic drug monitoring of imatinib in chronic myeloid leukemia: experience from 1216 patients at a centralized laboratory. Fundam Clin Pharmacol. 2013;27(6):690‐697. doi:10.1111/fcp.12007 23113675

[74] Barratt DT , Cox HK , Menelaou A , et al. CYP2C8 genotype significantly alters imatinib metabolism in chronic myeloid leukaemia patients. Clin Pharmacokinet. 2017;56(8):977‐985. doi:10.1007/s40262-016-0494-0 27995529

[75] Widmer N , Decosterd LA , Leyvraz S , et al. Relationship of imatinib‐free plasma levels and target genotype with efficacy and tolerability. Br J Cancer. 2008;98(10):1633‐1640. doi:10.1038/sj.bjc.6604355 18475296 PMC2391118

[76] Guilhot F , Hughes TP , Cortes J , et al. Plasma exposure of imatinib and its correlation with clinical response in the tyrosine kinase inhibitor optimization and selectivity trial. Haematologica. 2012;97(5):731‐738. doi:10.3324/haematol.2011.045666 22315495 PMC3342976

[77] Takahashi N , Wakita H , Miura M , et al. Correlation between imatinib pharmacokinetics and clinical response in Japanese patients with chronic‐phase chronic myeloid leukemia. Clin Pharmacol Ther. 2010;88(6):809‐813. doi:10.1038/clpt.2010.186 20980997

[78] Malhotra H , Sharma P , Bhargava S , Rathore OS , Malhotra B , Kumar M . Correlation of plasma trough levels of imatinib with molecular response in patients with chronic myeloid leukemia. Leuk Lymphoma. 2014;55(11):2614‐2619. doi:10.3109/10428194.2014.885515 24446903

[79] Singh N , Kumar L , Meena R , Velpandian T . Drug monitoring of imatinib levels in patients undergoing therapy for chronic myeloid leukaemia: comparing plasma levels of responders and non‐responders. Eur J Clin Pharmacol. 2009;65(6):545‐549. doi:10.1007/s00228-009-0621-z 19214491

[80] Awidi A , Ayed AO , Bsoul N , et al. Relationship of serum imatinib trough level and response in CML patients: long term follow‐up. Leuk Res. 2010;34(12):1573‐1575. doi:10.1016/j.leukres.2010.07.014 20688395

[81] Ishikawa Y , Kiyoi H , Watanabe K , et al. Trough plasma concentration of imatinib reflects BCR‐ABL kinase inhibitory activity and clinical response in chronic‐phase chronic myeloid leukemia: a report from the BINGO study. Cancer Sci. 2010;101(10):2186‐2192. doi:10.1111/j.1349-7006.2010.01643.x 20608939 PMC11158257

[82] Francis J , Dubashi B , Sundaram R , Pradhan SC , Chandrasekaran A . A study to explore the correlation of ABCB1, ABCG2, OCT1 genetic polymorphisms and trough level concentration with imatinib mesylate‐induced thrombocytopenia in chronic myeloid leukemia patients. Cancer Chemother Pharmacol. 2015;76(6):1185‐1189. doi:10.1007/s00280-015-2905-6 26546461

[83] Adattini JA , Adiwidjaja J , Gross AS , McLachlan AJ . Application of physiologically‐based pharmacokinetic modelling to understand real‐world outcomes in patients receiving imatinib for chronic myeloid leukaemia. Submitted, under Review. 2022. 10.1002/prp2.1082 PMC10326685 37417254

[84] Shin H , Choi SY , Kee KM , et al. Comprehensive analyses of safety and efficacy toward individualizing imatinib dosage in patients with chronic myeloid leukemia. Int J Hematol. 2020;111(3):417‐426. doi:10.1007/s12185-019-02805-9 31863342

[85] Vinay K , Yanamandra U , Dogra S , et al. Long‐term mucocutaneous adverse effects of imatinib in Indian chronic myeloid leukemia patients. Int J Dermatol. 2018;57(3):332‐338. doi:10.1111/ijd.13852 29266186

[86] Belsey SL , Ireland R , Lang K , et al. Women administered standard dose imatinib for chronic myeloid leukemia have higher dose‐adjusted plasma imatinib and norimatinib concentrations than men. Ther Drug Monit. 2017;39(5):499‐504. doi:10.1097/ftd.0000000000000440 28767619

[87] Li QB , Chen C , Chen ZC , et al. Imatinib plasma trough concentration and its correlation with characteristics and response in Chinese CML patients. Acta Pharmacol Sin. 2010;31(8):999‐1004. doi:10.1038/aps.2010.79 20644548 PMC4007812

[88] Touma JA , McLachlan AJ , Gross AS . The role of ethnicity in personalized dosing of small molecule tyrosine kinase inhibitors used in oncology. Transl Cancer Res. 2017;6(suppl 10):S1558‐S1591. doi:10.21037/tcr.2017.09.09

[89] Chuah CT , Nakamae H , Shen ZX , Bradley‐Garelik MB , Kim DW . Efficacy and safety of dasatinib versus imatinib in the east Asian subpopulation of the DASISION trial of newly diagnosed chronic myeloid leukemia in chronic phase. Leuk Lymphoma. 2014;55(9):2093‐2100. doi:10.3109/10428194.2013.866663 24289108 PMC4196520

[90] Hochhaus A , Saglio G , Hughes TP , et al. Long‐term benefits and risks of frontline nilotinib vs imatinib for chronic myeloid leukemia in chronic phase: 5‐year update of the randomized ENESTnd trial. Leukemia. 2016;30(5):1044‐1054. doi:10.1038/leu.2016.5 26837842 PMC4858585

[91] Nakamae H , Fukuda T , Nakaseko C , et al. Nilotinib vs. imatinib in Japanese patients with newly diagnosed chronic myeloid leukemia in chronic phase: long‐term follow‐up of the Japanese subgroup of the randomized ENESTnd trial. Int J Hematol. 2018;107(3):327‐336. doi:10.1007/s12185-017-2353-7 29076005

[92] Saussele S , Krauss MP , Hehlmann R , et al. Impact of comorbidities on overall survival in patients with chronic myeloid leukemia: results of the randomized CML study IV. Blood. 2015;126(1):42‐49. doi:10.1182/blood-2015-01-617993 25918346 PMC4574015

[93] Fogliatto L , Capra M , Schaan M , et al. Impact of comorbidity in event‐free survival, toxicity and adherence to treatment in chronic myeloid leukemia patients treated with imatinib. Blood 2010;116(21):2296. Accessed October 10, 2020. doi:10.1182/blood.V116.21.2296.2296

[94] Breccia M , Luciano L , Latagliata R , et al. Age influences initial dose and compliance to imatinib in chronic myeloid leukemia elderly patients but concomitant comorbidities appear to influence overall and event‐free survival. Blood. 2011;118(21):2751. doi:10.1182/blood.V118.21.2751.2751 25047978

[95] Gora‐Tybor J , Sacha T , Waclaw J , et al. Concomitant medications and comorbidities affect early response in CML patients treated with imatinib: a retrospective analysis of 340 patients from the polish adult leukemia group (PALG) registry. Blood. 2017;130(suppl 1):2887. doi:10.1182/blood.V130.Suppl_1.2887.2887

[96] Kunbaz A , Eşkazan AE , Ozmen D , et al. Potential drug‐drug interactions among patients with chronic myeloid leukemia under tyrosine kinase inhibitor therapy with imatinib: PB1929. HemaSphere. 2019;3:877. doi:10.1097/01.HS9.0000566212.35482.b2

[97] Whirl‐Carrillo M , McDonagh EM , Hebert JM , et al. Pharmacogenomics knowledge for personalized medicine. Clin Pharmacol Ther. 2012;92(4):414‐417. doi:10.1038/clpt.2012.96 22992668 PMC3660037

[98] Osorio S , Escudero‐Vilaplana V , Gómez‐Centurión I , González‐Arias E , García‐González X , Díez JL . Inadequate response to imatinib treatment in chronic myeloid leukemia due to a drug interaction with phenytoin. J Oncol Pharm Pract. 2017;25(3):694‐698. doi:10.1177/1078155217743565 29199506

[99] Taguchi K , Kouroki M , Ohmura T , Jono H , Endo F , Saito H . Carbamazepine‐imatinib interaction in a child with chronic myeloid leukemia. Pediatr Int 2014;56(4):e33‐6. Accessed January 10, 2019. 10.1111/ped.12382 25252068

[100] Wen PY , Yung WK , Lamborn KR , et al. Phase I/II study of imatinib mesylate for recurrent malignant gliomas: north American brain tumor consortium study 99‐08. Clin Cancer Res. 2006;12(16):4899‐4907. doi:10.1158/1078-0432.ccr-06-0773 16914578

[101] Pursche S , Schleyer E , von Bonin M , et al. Influence of enzyme‐inducing antiepileptic drugs on trough level of imatinib in glioblastoma patients. Curr Clin Pharmacol. 2008;3(3):198‐203. doi:10.2174/157488408785747656 18781906 PMC2748699

[102] Filppula AM , Neuvonen M , Laitila J , Neuvonen PJ , Backman JT . Autoinhibition of CYP3A4 leads to important role of CYP2C8 in imatinib metabolism: variability in CYP2C8 activity may alter plasma concentrations and response. Drug Metab Dispos. 2013;41(1):50‐59. doi:10.1124/dmd.112.048017 23028140

[103] O'Brien SG , Meinhardt P , Bond E , et al. Effects of imatinib mesylate (STI571, Glivec) on the pharmacokinetics of simvastatin, a cytochrome p450 3A4 substrate, in patients with chronic myeloid leukaemia. Br J Cancer. 2003;89(10):1855‐1859. doi:10.1038/sj.bjc.6601152 14612892 PMC2394453

[104] Bleyzac N , Kebaili K , Mialou V , Bertrand Y , Goutelle S . Pharmacokinetic drug interaction between cyclosporine and imatinib in bone marrow transplant children and model‐based reappraisal of imatinib drug interaction profile. Ther Drug Monit. 2014;36(6):724‐729. doi:10.1097/ftd.0000000000000084 24739665

[105] Atiq F , Broers AE , Andrews LM , et al. A clinically relevant pharmacokinetic interaction between cyclosporine and imatinib. Eur J Clin Pharmacol. 2016;72(6):719‐723. doi:10.1007/s00228-016-2038-9 26965514 PMC4865545

[106] Yang X , Bai Y , Shi M , et al. Validation of the EUTOS long‐term survival score in chinese chronic myeloid leukemia patients treated with imatinib: a multicenter real‐world study. Cancer Manag Res. 2020;12:1293‐1301. doi:10.2147/cmar.S237467 32110103 PMC7039071

[107] Geelen IGP , Sandin F , Thielen N , et al. Validation of the EUTOS long‐term survival score in a recent independent cohort of "real world" CML patients. Leukemia. 2018;32(10):2299‐2303. doi:10.1038/s41375-018-0136-7 29743721

[108] Millot F , Guilhot J , Suttorp M , et al. Prognostic discrimination based on the EUTOS long‐term survival score within the international registry for chronic myeloid leukemia in children and adolescents. Haematologica. 2017;102(10):1704‐1708. doi:10.3324/haematol.2017.170035 28838993 PMC5622854

[109] Castagnetti F , Gugliotta G , Breccia M , et al. The use of EUTOS long‐term survival score instead of Sokal score is strongly advised in elderly chronic myeloid leukemia patients. Blood. 2018;132(suppl 1):44. Accessed October 10, 2020. 10.1182/blood-2018-99-117409

[110] Bonifacio M , Scaffidi L , Binotto G , et al. Predictive factors of stable deep molecular response in chronic myeloid leukemia patients treated with imatinib standard dose: a study from the Gruppo Triveneto LMC. Blood 2015;126(23):597. Accessed May 10, 2019. 10.1182/blood.V126.23.597.597 25926600

[111] Lucas CM , Harris RJ , Giannoudis A , et al. Chronic myeloid leukemia patients with the e13a2 BCR‐ABL fusion transcript have inferior responses to imatinib compared to patients with the e14a2 transcript. Haematologica. 2009;94(10):1362‐1367. doi:10.3324/haematol.2009.009134 19713230 PMC2754951

[112] Hanfstein B , Lauseker M , Hehlmann R , et al. Distinct characteristics of e13a2 versus e14a2 BCR‐ABL1 driven chronic myeloid leukemia under first‐line therapy with imatinib. Haematologica. 2014;99(9):1441‐1447. doi:10.3324/haematol.2013.096537 24837466 PMC4562532

[113] Jain P , Kantarjian H , Patel KP , et al. Impact of BCR‐ABL transcript type on outcome in patients with chronic‐phase CML treated with tyrosine kinase inhibitors. Blood. 2016;127(10):1269‐1275. doi:10.1182/blood-2015-10-674242 26729897 PMC4786836

[114] Castagnetti F , Gugliotta G , Breccia M , et al. The BCR‐ABL1 transcript type influences response and outcome in Philadelphia chromosome‐positive chronic myeloid leukemia patients treated frontline with imatinib. Am J Hematol. 2017;92(8):797‐805. doi:10.1002/ajh.24774 28466557

[115] Lin HX , Sjaarda J , Dyck J , et al. Gender and BCR‐ABL transcript type are correlated with molecular response to imatinib treatment in patients with chronic myeloid leukemia. Eur J Haematol. 2016;96(4):360‐366. doi:10.1111/ejh.12597 26059983

[116] Mourad N , Kihel I , Guella D , et al. Sex and major molecular response to imatinib treatment for patients with chronic myeloid leukemia. Biochem Pharmacol. 2019;8(1):263. doi:10.35248/2167-0501.19.8.263

[117] Cortes J , Giles F , O'Brien S , et al. Result of high‐dose imatinib mesylate in patients with Philadelphia chromosome‐positive chronic myeloid leukemia after failure of interferon‐alpha. Blood. 2003;102(1):83‐86. doi:10.1182/blood-2003-01-0025 12637317

[118] Kantarjian H , Talpaz M , O'Brien S , et al. High‐dose imatinib mesylate therapy in newly diagnosed Philadelphia chromosome‐positive chronic phase chronic myeloid leukemia. Blood. 2004;103(8):2873‐2878. doi:10.1182/blood-2003-11-3800 15070658

[119] Castagnetti F , Palandri F , Amabile M , et al. Results of high‐dose imatinib mesylate in intermediate Sokal risk chronic myeloid leukemia patients in early chronic phase: a phase 2 trial of the GIMEMA CML working party. Blood. 2009;113(15):3428‐3434. doi:10.1182/blood-2007-08-103499 19211938

[120] Hughes TP , Branford S , White DL , et al. Impact of early dose intensity on cytogenetic and molecular responses in chronic‐ phase CML patients receiving 600 mg/day of imatinib as initial therapy. Blood. 2008;112(10):3965‐3973. doi:10.1182/blood-2008-06-161737 18768781

[121] Koh Y , Kim I , Yoon SS , et al. Phase IV study evaluating efficacy of escalated dose of imatinib in chronic myeloid leukemia patients showing suboptimal response to standard dose imatinib. Ann Hematol. 2010;89(7):725‐731. doi:10.1007/s00277-010-0910-8 20179930

[122] Deininger MW , Kopecky KJ , Radich JP , et al. Imatinib 800 mg daily induces deeper molecular responses than imatinib 400 mg daily: results of SWOG S0325, an intergroup randomized PHASE II trial in newly diagnosed chronic phase chronic myeloid leukaemia. Br J Haematol. 2014;164(2):223‐232. doi:10.1111/bjh.12618 24383843 PMC4127316

[123] Michel C , Burchert A , Hochhaus A , et al. Imatinib dose reduction in major molecular response of chronic myeloid leukemia: results from the German chronic myeloid leukemia‐study IV. Haematologica. 2019;104(5):955‐962. doi:10.3324/haematol.2018.206797 30514803 PMC6518910

[124] Cervantes F , Correa JG , Pérez I , et al. Imatinib dose reduction in patients with chronic myeloid leukemia in sustained deep molecular response. Ann Hematol. 2017;96(1):81‐85. doi:10.1007/s00277-016-2839-z 27717993

[125] Latagliata R , Carmosino I , Vozella F , et al. Impact of exclusion criteria for the DASISION and ENESTnd trials in the front‐line treatment of a ‘real‐life’ patient population with chronic myeloid leukaemia. Hematol Oncol. 2017;35(2):232‐236. doi:10.1002/hon.2274 26648184

[126] Australian Institute of Health and Welfare . Cancer Data in Australia. Australian Institute of Health and Welfare; 2020. Accessed November 13, 2020. https://www.aihw.gov.au/reports/cancer/cancer‐data‐in‐australia

