# Supplementary material for: Real‐world efficacy and safety outcomes of imatinib treatment in patients with chronic myeloid leukemia: An Australian experience
Source: Pharmacol Res Perspect. 2022 Sep 14;10(5):e01005. doi: 10.1002/prp2.1005 (PMC9475133; doi:10.1002/prp2.1005)
Supplement: Supplementary file 1 — Appendix S1 [file PRP2-10-e01005-s001.docx]

**ONLINE SUPPLEMENTARY INFORMATION**

Real-world efficacy and safety outcomes of imatinib treatment in patients with chronic myeloid leukemia: an Australian experience

Josephine A Adattini, Annette S Gross, Nicole Wong Doo, Andrew J McLachlan

SUPPLEMENTARY METHODS

***Ethics approval***

Approval of the study protocol was obtained through the Sydney Local Health District Human Research Ethics Committee (reference: LNR/17/CRGH/248) and included a waiver of informed consent in line with the requirements of the National Statement on Ethical Conduct in Human Research.

***Definitions of variables and endpoints***

Demographic and disease characteristics

Hepatic function was defined using the National Cancer Institute–Organ Dysfunction Working Group Hepatic Impairment Criteria.^1,2^ Kidney function was defined using the Kidney Disease: Improving Global Outcomes Chronic Kidney Disease Guideline.^3^ Poorly controlled diabetes was defined as a glycosylated haemoglobin (HbA1c) > 7%.^4^ Poorly controlled hypertension was defined as blood pressure ≥ 140/90 mmHg.^5^ Physician reported Eastern Cooperative Oncology Group (ECOG) Performance Status (PS)^6^ at diagnosis was extracted from haematology databases. If ECOG PS was not documented in the haematology database, it was determined by reviewing medical notes at diagnosis.

Prescribing patterns

Commencing treatment with imatinib for newly diagnosed chronic myeloid leukemia (CML) was classified as first-line treatment, whereas receiving imatinib due to resistance or intolerance to previous CML treatments was classified as second-line or later. Imatinib treatment discontinuation was defined as having a gap of more than 120 days with no CML treatment after the last imatinib administration, having a subsequent CML treatment after the initial imatinib regimen, or death (of any cause) while on imatinib treatment. Time to discontinuation (TTD) was defined as the length of time from imatinib initiation until discontinuation, with patients who did not discontinue imatinib censored at the end of follow-up. Time to next line of treatment (TTNT) was defined as the time from imatinib initiation until commencement of another line of tyrosine kinase inhibitor (TKI) treatment for CML or death, whichever came first. When subsequent treatment was not received (e.g., continuing imatinib or discontinued with no further treatments [not because of death]), patients were censored at end of follow-up. Time to first dose modification was defined as the length of time from the date of imatinib treatment initiation until the first imatinib dose modification. A dose modification included dose reduction, dose escalation, dose interval changes and temporary treatment interruption. Patients who did not require a dose modification were censored at the end of treatment follow-up.

Efficacy outcomes

Molecular response (MR) endpoints were defined using quantitative *BCR-ABL1* transcript levels. *BCR-ABL1* transcript levels of ≤ 0.1%, ≤0.01%, ≤0.0032%, ⩽0.001% on the international scale (IS) or ≥3 log, ≥4 log, ≥4.5 and ≥5.0 log reduction of *BCR-ABL1* mRNA transcripts from baseline (in molecular laboratories not able to report on the IS at the time) were defined major molecular response (MMR), MR^4.0^, MR^4.5^ and MR^5.0^ respectively.^7^ MR^4.0^, MR^4.5^ and MR^5.0^ are classified as deep molecular response (DMR).^7^ Undetectable *BCR-ABL1* transcript levels were classified as a MMR when control gene numbers were not available to determine the sensitivity of the test. Achievement of sDMR (sustained DMR; DMR maintained for at least 2 consecutive years) and Early Molecular Response (EMR; BCR-ABL1IS ≤ 10% at 3 and 6 months) were also documented. Relapse was defined as a composite endpoint of; loss of MMR, loss of haematological or cytogenetic response, progression to accelerated/blast phase, or death due to CML.^7^

Tolerability outcomes

Medical management of adverse events were recorded, and included imatinib dose adjustments, imatinib treatment discontinuation, commencement of short-term or long-term medicines, changes in existing concomitant medicines, and increased healthcare visits. Short-term medicines were defined as medicines used for symptomatic management of adverse drug reaction (ADR) episodes, including analgesics, antibiotics, antiemetics, antacids, diuretics, supplements to correct electrolyte imbalances and blood transfusions. Changes in long-term medicine regimens included dose changes to existing medicines, or the commencement of new medicines used to manage comorbidities arising from imatinib-related ADRs (e.g., lipid lowering agents for hypercholesterolemia, thyroxine for hypothyroidism, antihypertensives and beta-blockers for cardiovascular complications, antiplatelets or anticoagulants for treatment of embolic events, and inhalers for respiratory complications).

Considering clinical trial exclusion criteria

The ENESTnd (Evaluating Nilotinib Efficacy and Safety in Clinical Trials–Newly Diagnosed Patients) trial excluded patients with an ECOG PS ≥ 3, with impaired cardiac function, with severe or uncontrolled medical conditions (including uncontrolled diabetes, active or uncontrolled infection), receiving concomitant treatment with a therapeutic coumarin derivative (e.g., warfarin), receiving concomitant treatment with medicines known to inhibit or induce CYP3A4 or medicines with the potential to prolong the QTc interval.^8^

The DASISION (Dasatinib versus Imatinib Study In treatment-Naive CML patients) trial excluded patients with an ECOG PS ≥ 3, with uncontrolled or serious medical disorders (including cardiovascular disease) or active infections, inadequate hepatic or renal function, a corrected QTc interval > 450 milliseconds, a history of a serious bleeding disorder unrelated to CML, previous or concurrent cancer (other than basal-cell skin cancer), and pleural effusion at baseline.^9^

The number of patients with baseline comorbidities and concomitant medicines listed as exclusion criteria in the ENESTnd and DASISION clinical trials are presented in Supplementary Table 1. ENESTnd and DASISION clinical trial exclusion criteria known not to reflect risks with imatinib treatment (e.g., receiving concomitant treatment with QTc interval prolonging medicines) were omitted from clinical trial eligibility categorisation (eligible *vs.* ineligible) in this study.

***Time to first event analyses using the Kaplan-Meier method and Cox-proportional hazards regression***

The Kaplan-Meier method^10^ was used to estimate the time to first dose modification, TTD, TTNT, and real-world survival endpoints (event-free survival [EFS], progression-free survival [PFS], overall survival [OS]). To compare time-to-event of these endpoints between two or more groups, a Cox-proportional hazards (Cox) model was used.^11^ Data are reported as unadjusted Hazard Ratios (HRs) with associated 95% confidence intervals (CIs). Any variables that suggested an effect on the hazard of EFS in univariable regression (a significance level of α=0.10 and clinical plausibility) were then used in multivariable regression, with a forward stepwise variable selection method using the Akaike Information Criterion employed to find the best model.^12^ Data are reported as adjusted Hazard Ratios (HRs) with associated 95% CIs.

***Cumulative incidence with competing events***

The Cumulative Incidence Competing Risk method^13-15^ was used to model the cumulative incidences of molecular response and imatinib-related ADRs, adjusting for competing risks and different follow-up periods. For those who experienced an event (molecular response, ADR), the date used was the date associated with the first occurrence of the event. Those who did not experience the event, but discontinued treatment or died whilst on treatment (competing risks), were censored at the time of discontinuation or death (whichever occurred first). Patients who did not experience the event but were still on treatment, were censored at the end of treatment follow-up (or censored at the date of the last *BCR-ABL1* reading, if the event was molecular response). The cumulative incidence of molecular response was only calculated among evaluable patients who had valid molecular monitoring at baseline and during TKI treatment. The cumulative incidence of an ADR was calculated among all study patients. Cumulative incidence curves are presented in the results to model the cumulative incidence of these outcome variables.

To examine the effect of patient and treatment characteristics on the cumulative incidence of molecular response and ADRs (all grade, grade ≥3), univariable regression analysis were conducted using the Fine-Gray Subdistribution hazard model, which also takes into account competing risks.^16-20^ Unadjusted Subdistribution Hazard Ratios (SHRs) and associated 95% CIs are reported. Any variables that suggested an effect on the cumulative incidence of molecular response and or ADRs in univariable regression (a significance level of α=0.10 and clinical plausibility) were then used in a multivariable regression, with a forward stepwise variable selection method using the Akaike Information Criterion employed to find the best model.^12^ Adjusted SHRs and associated 95% CIs are reported.

***Beyond time-to first event: analyses of recurrent event outcomes***

To evaluate the effect of patient and treatment characteristics on the risk of recurrent imatinib-related ADRs, univariable regression analyses were conducted using the Prentice, Williams and Peterson Total Time (PWP-TT) model.^21^ Data from univariable regression analyses are presented as unadjusted HRs and their corresponding 95% CIs. Any variables that suggested an effect on the HR of recurrent ADRs in univariable regression (a significance level of α=0.10 and clinical plausibility) were then used in a multivariable PWP-TT analysis. A forward stepwise variable selection method was employed to find the best model, using the Akaike Information Criterion.^12^ Data are presented as adjusted HRs and their corresponding 95% CIs.

SUPPLEMENTARY TABLES

Supplementary Table 1: Reason(s) for likely ineligibility for inclusion in controlled clinical trials

| **Eligibility criteria for ENESTnd^8^ and DASISION^9^ clinical trials** | **Number of patients** |
| --- | --- |
| ECOG PS ≥ 3 | 1 |
| Pre-existing impaired cardiac function | 21 |
| Pre-existing severe or uncontrolled medical conditions | 44 |
| Pre-existing hepatic or kidney dysfunction | 18 |
| Pre-existing corrected QTc interval > 450 milliseconds | 0 |
| Pre-existing pleural effusion | 0 |
| A history of a serious bleeding disorder unrelated to CML | 0 |
| Previous or concurrent cancer (other than basal-cell skin cancer) | 0 |
| Receiving concomitant treatment with medicines known to inhibit or induce CYP3A4 | 7 |
| Receiving concomitant treatment with a therapeutic coumarin derivative | 0 |
| Receiving concomitant treatment with medicines known to prolong the QTc interval | 0 |

CYP, cytochrome P450; ECOG PS, Eastern Cooperative Oncology Group Performance Status

Supplementary Table 2: Baseline demographic and CML disease characteristics of the imatinib treated cohort compared by line of treatment

| **Characteristics** | | **Line of treatment** | | |
| --- | --- | --- | --- | --- |
|  |  | **First-line** | **Second-line or later** | ***P* Value*** |
|  |  | **(N=78)** | **(N=8)** |  |
| ***Age at diagnosis (years), mean (SD)*** | | 55 (18) | 56 (9) | 0.91 |
| ***CCI score, median (range; IQR)*** | | 4 (2–12; 2–5.8) | 3 (2–9; 3–3.5) | 0.59 |
| ***Male, n (%)*** | | 48 (62) | 3 (38) | 0.26 |
| ***Geographic ancestry^a^, n (%)*** | European | 60 (77) | 4 (50) | <0.05* |
|  | East Asian | 8 (10) | 4 (50) |  |
|  | South Asian | 4 (5) | 0 |  |
|  | Other^b^ | 6 (8) | 0 |  |
| ***Comorbidities at diagnosis, n (%)*** | Cardiovascular disease | 19 (24) | 2 (25) | 1 |
|  | Poorly controlled diabetes | 13 (17) | 0 | 0.60 |
|  | Poorly controlled hypertension | 9 (12) | 3 (38) | 0.08 |
|  | Chronic pulmonary disease | 11 (14) | 0 | 0.59 |
|  | Peripheral vascular disease | 9 (12) | 1 (13) | 1 |
|  | Hypothyroidism post thyroidectomy | 3 (4) | 1 (13) | 0.33 |
|  | History of pancreatitis | 2 (3) | 0 | 1 |
|  | Cerebrovascular disease | 2 (3) | 0 | 1 |
|  | None of the above | 40 (51) | 2 (25) | 0.27 |
| ***Family history of cardiovascular disease, n (%)*** | Yes | 24 (56) | 1 (20) | 0.18 |
|  | No | 19 (44) | 4 (80) |  |
|  | *Unknown* | *35* | *3* |  |
| ***Concomitant medicines, n (%)*** | CYP3A4 substrate | 42 (54) | 3 (38) | 0.47 |
|  | Antiplatelet | 37 (47) | 3 (38) | 0.72 |
|  | Paracetamol | 10 (13) | 0 | 0.59 |
|  | Antineoplastic | 9 (12) | 0 | 0.59 |
|  | Digoxin | 6 (8) | 1 (13) | 0.51 |
|  | Thyroxine | 5 (6) | 1 (13) | 0.45 |
|  | CYP2C8 inhibitor | 6 (8) | 0 | 1 |
|  | P-gp inhibitor | 5 (6) | 0 | 1 |
|  | CYP3A4 inhibitor | 3 (4) | 1 (13) | 0.13 |
|  | CYP3A4 inhibitor, CAM | 2 (3) | 1 (13) |  |
|  | CYP3A4 inducer | 1 (1) | 0 | 1 |
|  | CYP3A4 inducer, CAM | 1 (1) | 0 |  |
|  | None of the above | 22 (28) | 3 (38) | 0.69 |
| ***Disease phase, n (%)*** | Chronic | 71 (91) | 7 (88) | 0.56 |
|  | Accelerated | 7 (9) | 1 (13) |  |
| ***Extramedullary leukaemia present, n (%)*** | | 0 | 1 (13) | 0.09 |
| ***ECOG PS, n (%)*** | ECOG PS 0 | 47 (60) | 5 (63) | 0.09^c^ |
|  | ECOG PS 1 | 26 (33) | 3 (38) |  |
|  | ECOG PS 2 | 4 (5) | 0 |  |
|  | ECOG PS 3 | 0 | 0 |  |
|  | ECOG PS 4 | 1 (1) | 0 |  |
| ***Sokal score, n (%)*** | Low | 17 (24) | 2 (25) | 1 |
|  | Intermediate | 36 (50) | 4 (50) |  |
|  | High | 19 (26) | 2 (25) |  |
|  | *Unknown* | *6* | *0* |  |
| ***ELTS score, n (%)*** | Low | 35 (49) | 5 (62.5) | 0.90 |
|  | Intermediate | 24 (33) | 2 (25) |  |
|  | High | 13 (18) | 1 (12.5) |  |
|  | *Unknown* | *6* | *0* |  |
| ***Additional BM karyotype abnormalities, n (%)*** | Yes | 6 (11) | 0 | 1 |
|  | No | 47 (89) | 7 (100) |  |
|  | *Unknown* | *25* | *1* |  |
| ***BM fibrosis, n (%)*** | Yes | 32 (78) | 3 (60) | 0.58 |
|  | No | 9 (22) | 2 (40) |  |
|  | *Unknown* | *37* | *3* |  |
| ***BCR-ABL1 transcript type, n (%)*** | e13a2 (b2a2) | 22 (39) | 5 (71) | 0.46^d^ |
|  | e14a2 (b3a2) | 16 (28) | 0 |  |
|  | e13a2 (b2a2) and e14a2 (b3a2) | 6 (11) | 1 (14) |  |
|  | e13a2 (b2a2) and e1a2 | 6 (11) | 0 |  |
|  | e14a2 (b3a2) and e1a2 | 3 (5) | 1 (14) |  |
|  | e1a2 | 2 (4) | 0 |  |
|  | e19a2 | 1 (2) | 0 |  |
|  | e12a2, e14a2 (b3a2) and e1a2 | 1 (2) | 0 |  |
|  | *Unknown* | *21* | *1* |  |

BM, bone marrow; CAM, complementary or alternative medicine; CCI, Charlson Comorbidity Index; CYP, cytochrome P450; ECOG PS, Eastern Cooperative Oncology Group Performance Status; ELTS, European Treatment and Outcome Study (EUTOS) long-term survival; IQR, interquartile range; P-gp, P-glycoprotein; SD, standard deviation

** Statistically significant difference (α <0.05). Quantitative variables evaluated using the independent two sample t-test or Wilcoxon-Mann-Whitney test. Categorical variables evaluated using Pearson’s chi-squared test of independence or Fisher’s exact test of independence.*

*^a^ Geographic ancestry was assigned using information contained on patient registration forms and in medical records.*

*^b^ 3 individuals of Middle Eastern/North African ancestry (1 Lebanon, 1 Iran, 1 Egypt) and 3 of Pacific Islander ancestry (Maori).*

*^c^ Difference between groups also not statistically significant if comparing ECOG PS of 0, 1 and 2 or more (P = 1)*

*^d^ comparison between e13a2, e14a2, e13a2 with e14a2, and other.*

Supplementary Table 3: Cumulative incidence of imatinib-related grade ≥3 adverse drug reactions (ADRs) and the hazard of recurrent grade ≥3 ADRs according to baseline characteristics

| **Characteristic** | **Level** | **No. of patients** | **No. patients who had a grade ≥3 ADR** | **Cumulative incidence of a grade ≥3 ADR at 18 months, % (95% CI)^a^** | **Unadjusted SHR (95% CI) of a grade ≥3 ADR^b^** | ***P* Value*** | **Unadjusted HR (95% CI) of grade ≥3 ADR recurrence^c^** | ***P* Value*** |
| --- | --- | --- | --- | --- | --- | --- | --- | --- |
| ***Line of treatment*** | Second-line or later | 11 | 9 | 76 (29–94) | 1.70 (0.80–3.59) | 0.17 | 1.05 (0.78–1.40) | 0.76 |
|  | First-line | 78 | 56 | 50 (39–61) | Reference |  | Reference |  |
| ***Monotherapy*** | No | 5 | 5 | 100 | 3.65 (2.02–6.57) | <0.001* | 1.40 (0.82–2.40) | 0.22 |
|  | Yes | 84 | 60 | 50 (39–61) | Reference |  | Reference |  |
| ***Imatinib starting dose*** | 400 or 500 mg/day | 46 | 29 | 42 (27–56) | 0.54 (0.33–0.87) | <0.05* | 0.82 (0.60–1.13) | 0.17 |
|  | 600 or 800 mg/day | 43 | 36 | 65 (49–78) | Reference |  | Reference |  |
| ***Sex*** | Female | 36 | 28 | 61 (43–75) | 1.23 (0.75–2.01) | 0.41 | 1.33 (1.01–1.77) | <0.05* |
|  | Male | 53 | 37 | 48 (34–61) | Reference |  | Reference |  |
| ***Age at imatinib initiation (10 years)*** | | 89 | 65 | - | 1.13 (0.96–1.32)^e^ | 0.15 | 1.10 (0.98–1.23)^e^ | <0.10* |
| ***Weight at imatinib initiation (10 kg)^d^*** | | 74 | 54 | - | 0.88 (0.75–1.03)^e^ | <0.10* | 0.89 (0.81–0.98)^e^ | <0.05* |
| ***Geographic ancestry ^f^*** | East Asian | 14 | 12 | 71 (38–89) | 1.85 (0.93–3.68) | 0.22 | 1.13 (0.76–1.68) | <0.10* |
|  | South Asian | 4 | 3 | 75 (2–98) | 1.33 (0.34–5.27) |  | 1.81 (0.96–3.43) |  |
|  | Other^g^ | 6 | 5 | 83 (9–99) | 2.34 (0.66–8.30) |  | 1.59 (0.83–3.02) |  |
|  | European | 65 | 45 | 45 (33–57) | Reference |  | Reference |  |
| ***CCI score at diagnosis*** | | 89 | 65 | - | 1.11 (1.01–1.22) | <0.05* | 1.10 (1.05–1.16) | <0.001* |
| ***ECOG PS*** | | 89 | 65 | - | 1.17 (0.83–1.65) | 0.38 | 1.29 (1.10–1.51) | <0.05* |
| ***Concomitant medicines with potential for drug interactions^h^*** | Yes | 62 | 50 | 80 (67-89) | 1.91 (1.05–3.48) | <0.05* | 1.46 (1.02–2.10) | <0.05* |
|  | No | 27 | 15 | 50 (29–68) | Reference |  | Reference |  |
| ***Pre-existing cardiovascular disease*** | Yes | 21 | 17 | 59 (33–77) | 1.38 (0.83–2.28) | 0.13 | 1.52 (1.13–2.05) | <0.05* |
|  | No | 68 | 48 | 52 (39–63) | Reference |  | Reference |  |
| ***Pre-existing chronic pulmonary disease*** | Yes | 12 | 10 | 75 (35–92) | 1.45 (0.73–2.89) | 0.33 | 1.59 (1.04–2.43) | <0.05* |
|  | No | 77 | 55 | 50 (38–60) | Reference |  | Reference |  |
| ***Pre-existing poorly controlled hypertension*** | Yes | 12 | 8 | 43 (14–69) | 0.94 (0.46–1.92) | 0.86 | 1.12 (0.72–1.72) | 0.62 |
|  | No | 77 | 57 | 55 (43–65) | Reference |  | Reference |  |
| ***Pre-existing poorly controlled diabetes*** | Yes | 13 | 10 | 55 (23–79) | 1.35 (0.74–2.46) | 0.33 | 1.07 (0.73–1.55) | 0.74 |
|  | No | 76 | 53 | 53 (41–64) | Reference |  | Reference |  |
| ***Pre-existing peripheral vascular disease*** | Yes | 10 | 8 | 60 (22–84) | 1.16 (0.58–2.32) | 0.68 | 1.47 (1.06–2.04) | <0.05* |
|  | No | 79 | 57 | 52 (41–63) | Reference |  | Reference |  |
| ***Family history of cardiovascular disease^d^*** | Yes | 25 | 15 | 48 (27–66) | 0.53 (0.28–1.02) | <0.10* | 0.68 (0.42–1.08) | <0.10* |
|  | No | 24 | 20 | 63 (39–79) | Reference |  | Reference |  |

CCI, Charlson Comorbidity Index; CI, confidence interval; ECOG PS, Eastern Cooperative Oncology Group Performance Status; HR, Hazards Ratio; SHR, Subdistribution Hazards Ratio.

* Statistically significant difference (α <0.10)

^a^ Cumulative incidences are calculated using the cumulative incidence competing risk method.

^b^ Subdistribution Hazard Ratios are calculated using the Fine-Gray Subdistribution hazards model. This represents the unadjusted hazard of the first event.

^c^ Hazard ratios of recurrent events are calculated using the Prentice, Williams and Peterson Total Time model. This represents the unadjusted hazard.

^d^ Weight unknown in 15 patients (11 experienced a grade ≥3 ADR), family history of cardiovascular disease unknown in 40 patients (30 experienced a grade ≥3 ADR)

^e^ A 10-year increase in age is associated with a 13% increase in the Subdistribution hazard of a grade ≥3 ADR and a 10% increase in the hazard of recurrent grade ≥3 ADRs. A 10 kg increase in weight is associated with a 12% decrease in the Subdistribution hazard of a grade ≥3 ADR and an 11% decrease in the hazard of recurrent grade ≥3 ADRs.

^f^ Geographic ancestry was assigned using information contained on patient registration forms and in medical records.

^g^ 3 individuals of Middle Eastern/North African ancestry (1 Lebanon, 1 Iran, 1 Egypt) and 3 of Pacific Islander ancestry (Maori).

^h^ Concomitant medicines with the potential for an imatinib drug-drug interaction include CYP3A4 substrates, CYP3A4 inhibitors/inducers, CYP2C8 inhibitors, P-glycoprotein inhibitors, thyroxine, digoxin, paracetamol, antiplatelets and antineoplastic agents.

Supplementary Table 4: Cumulative incidence of major molecular response (MMR) in patients receiving imatinib treatment according to baseline characteristics

| **Characteristic** | **Level** | **No. of patients** | **No. achieved MMR** | **Unadjusted SHR (95% CI) of MMR** | ***P* Value*** |
| --- | --- | --- | --- | --- | --- |
| ***Line of treatment*** | Second-line or later | 7 | 6 | 1.25 (0.64–2.44) | 0.51 |
|  | First-line | 66 | 43 | Reference |  |
| ***Monotherapy*** | No | 2 | 1 | NE | NE^a^ |
|  | Yes | 71 | 48 | NE |  |
| ***Imatinib starting dose*** | 400 or 500 mg/day | 38 | 24 | 0.67 (0.39–1.18) | 0.16 |
|  | 600 or 800 mg/day | 35 | 25 | Reference |  |
| ***Sex*** | Female | 29 | 20 | 1.15 (0.65–2.05) | 0.63 |
|  | Male | 44 | 29 | Reference |  |
| ***Age at imatinib initiation (10 years)*** | - | 73 | 49 | 1.00 (0.86–1.16)^b^ | 0.97 |
| ***Weight at imatinib initiation (10 kg)*** | - | 60 | 39 | 1.01 (0.87–1.17)^b^ | 0.93 |
|  | *Unknown* | *13* | *10* | *–* | *–* |
| ***Geographic ancestry^c^*** | East Asian | 12 | 9 | 1.09 (0.54–2.17) | 0.68 |
|  | South Asian | 4 | 3 | 1.23 (0.38–4.00) |  |
|  | Other^d^ | 5 | 2 | 0.44 (0.10–1.85) |  |
|  | European | 52 | 35 | Reference |  |
| ***CCI score at diagnosis*** | - | 73 | 49 | 0.93 (0.83–1.04) | 0.21 |
| ***Disease phase*** | Accelerated | 2 | 1 | 0.62 (0.09–4.48) | 0.64 |
|  | Chronic | 71 | 48 | Reference |  |
| ***BCR-ABL1 transcript type, n (%)*** | e14a2 (b3a2) | 15 | 9 | 0.65 (0.31–1.44) | <0.001* |
|  | e13a2 (b2a2) and e14a2 (b3a2) | 5 | 4 | 3.17 (1.44–6.73) |  |
|  | Other ^e^ | 12 | 5 | 0.44 (0.15–1.33) |  |
|  | e13a2 (b2a2) | 23 | 18 | Reference |  |
|  | *Unknown* | *18* | *13* | *–* | *–* |
| ***BM fibrosis, n (%)*** | Yes | 29 | 19 | 1.17 (0.53–2.56) | 0.70 |
|  | No | 11 | 7 | Reference |  |
|  | *Unknown* | *33* | *23* | *–* | *–* |
| ***Additional BM karyotype abnormalities, n (%)*** | Yes | 4 | 3 | 1.39 (0.35–5.47) | 0.64 |
|  | No | 45 | 32 | Reference |  |
|  | *Unknown* | *24* | *14* | *–* | *–* |
| ***ECOG PS*** | - | 73 | 49 | 0.75 (0.49–1.14) | 0.18 |
| ***ELTS score*** | High | 10 | 3 | 0.17 (0.06–0.48) | <0.001* |
|  | Intermediate | 19 | 9 | 0.38 (0.17–0.83) |  |
|  | Low | 38 | 33 | Reference |  |
|  | *Unknown* | *6* | *4* | *–* | *–* |
| ***Sokal score*** | High | 15 | 8 | 0.39 (0.18–0.87) | <0.05* |
|  | Intermediate | 34 | 21 | 0.56 (0.30–1.04) |  |
|  | Low | 18 | 16 | Reference |  |
|  | *Unknown* | *6* | *4* | *–* | *–* |
| ***Concomitant medicines with potential for drug interactions ^f^*** | Yes | 51 | 37 | 1.92 (1.05–3.51) | <0.05* |
|  | No | 22 | 12 | Reference |  |
| ***Pre-existing cardiovascular disease*** | Yes | 17 | 11 | 0.86 (0.50–1.50) | 0.60 |
|  | No | 56 | 38 | Reference |  |
| ***Pre-existing chronic pulmonary disease*** | Yes | 11 | 6 | 0.57 (0.26–1.25) | 0.16 |
|  | No | 62 | 43 | Reference |  |
| ***Pre-existing poorly controlled hypertension*** | Yes | 9 | 7 | 1.17 (0.57–2.41) | 0.67 |
|  | No | 64 | 42 | Reference |  |
| ***Pre-existing poorly controlled diabetes*** | Yes | 11 | 6 | 0.76 (0.33–1.71) | 0.50 |
|  | No | 62 | 43 | Reference |  |
| ***Pre-existing peripheral vascular disease*** | Yes | 9 | 5 | 0.77 (0.28–2.06) | 0.60 |
|  | No | 64 | 44 | Reference |  |

BM, bone marrow; CCI, Charlson Comorbidity Index; CI, confidence interval; ECOG PS, Eastern Cooperative Oncology Group Performance Status; ELTS, EUTOS long-term survival; EUTOS, European Treatment and Outcome Study; NE, not evaluable; SHR, Subdistribution Hazard Ratio.

* Statistically significant difference (α <0.10) evaluated using univariable Fine-Gray subdistribution hazards regression.

^a^ Not evaluable due to the small sample of patients using imatinib in combination with another antineoplastic that were evaluable for and achieved MMR.

^b^ A 10-year increase in age is associated with no change in the Subdistribution hazard of MMR. A 10 kg increase in weight is associated with a 1% increase in the Subdistribution hazard of MMR. These changes are not statistically significant.

^c^ Geographic ancestry was assigned using information contained on patient registration forms and in medical records.

^d^ 2 individuals were of Middle Eastern/North African ancestry (1 Lebanese, 1 Egyptian) and 3 of Pacific Islander ancestry (Maori).

^e^ Other BCR-ABL1 transcripts: e14a2 with e1a2, e13a2 with e1a2, e1a2, e19a2, and combined e12a2, e14a2 with e1a2.

^f^ Concomitant medicines with the potential for an imatinib drug-drug interaction include CYP3A4 substrates, CYP3A4 inhibitors/inducers, CYP2C8 inhibitors, P-glycoprotein inhibitors, thyroxine, digoxin, paracetamol, antiplatelets and antineoplastic agents.

*Supplementary Table 5: Event-free survival (EFS) in patients receiving imatinib treatment, according to baseline characteristics*

| **Characteristic** | **Level** | **No. of patients** | **No. of events** ^a^ | **3-year EFS rate,**  **% (95% CI)** | **Unadjusted HR (95% CI) of an event** ^a^ | ***P* Value*** |
| --- | --- | --- | --- | --- | --- | --- |
| ***Line of treatment*** | Second-line or later | 11 | 4 | 75 (50–100) | 1.98 (0.65–5.97) | 0.20 |
|  | First-line | 78 | 16 | 82 (73–93) | Reference |  |
| ***Monotherapy*** | No | 5 | 3 | 38 (8–100) | 8.57 (2.31–31.80) | <0.001* |
|  | Yes | 84 | 17 | 84 (75–94) | Reference |  |
| ***Imatinib starting dose*** | 400 or 500 mg/day | 46 | 13 | 77 (65–93) | 1.47 (0.58–3.71) | 0.41 |
|  | 600 or 800 mg/day | 43 | 7 | 87 (75–100) | Reference |  |
| ***Sex*** | Female | 36 | 9 | 79 (64–98) | 1.20 (0.50–3.00) | 0.64 |
|  | Male | 53 | 11 | 82 (71–95) | Reference |  |
| ***Age at imatinib initiation (10 years)*** | | 89 | 20 | – | 1.04 (0.78–1.39)^c^ | 0.78 |
| ***Weight at imatinib initiation (10 kg)^b^*** | | 74 | 17 | – | 1.03 (0.78–1.34)^c^ | 0.86 |
| ***Geographic ancestry***^d^ | East Asian | 14 | 4 | 82 (61–100) | 1.2 (0.4–3.5) | 0.78 |
|  | South Asian | 4 | 0 | – | – |  |
|  | Other^e^ | 6 | 0 | – | – |  |
|  | European | 65 | 16 | 80 (69–92) | Reference |  |
| ***CCI score at diagnosis*** | | 89 | 20 | – | 1.14 (0.93–1.40) | 0.21 |
| ***Disease phase*** | Accelerated | 8 | 3 | 80 (52–100) | 2.40 (0.70–8.10) | 0.17 |
|  | Chronic | 81 | 17 | 81 (72–92) | Reference |  |
| ***BCR-ABL1 transcript type, n (%)^b^*** | e14a2 (b3a2) | 18 | 4 | 87 (72–100) | 0.71 (0.21–2.41) | 0.90 |
|  | e13a2 (b2a2) and e14a2 (b3a2) | 7 | 1 | 80 (52–100) | 0.46 (0.06–3.67) |  |
|  | Other^f^ | 14 | 2 | 72 (44–100) | 0.99 (0.20–4.81) |  |
|  | e13a2 (b2a2) | 27 | 8 | 81 (66–100) | Reference |  |
| ***BM fibrosis, n (%)^b^*** | Yes | 36 | 5 | 89 (78–100) | 0.60 (0.14–2.52) | 0.48 |
|  | No | 11 | 3 | 89 (71–100) | Reference |  |
| ***Additional BM karyotype abnormalities, n (%)^b^*** | Yes | 6 | 1 | 100 | 0.81 (0.10–6.30) | 0.81 |
|  | No | 55 | 10 | 90 (81–100) | Reference |  |
| ***ECOG PS*** | | 89 | 20 | – | 1.40 (0.70–2.70) | 0.39 |
| ***ELTS score^b^*** | High | 15 | 5 | 91 (75–100) | 2.35 (0.76–7.19) | 0.30 |
|  | Intermediate | 26 | 5 | 81 (64–100) | 1.70 (0.55–5.23) |  |
|  | Low | 42 | 8 | 80 (67–94) | Reference |  |
| ***Sokal score^b^*** | High | 22 | 9 | 73 (53–100) | 2.97 (0.91–9.73) | <0.05* |
|  | Intermediate | 42 | 5 | 89 (77–100) | 0.75 (0.18–3.07) |  |
|  | Low | 19 | 4 | 76 (57–100) | Reference |  |
| ***Concomitant medicines with potential for drug interactions^g^*** | Yes | 62 | 15 | 78 (66–91) | 1.60 (0.60–4.40) | 0.38 |
|  | No | 27 | 5 | 90 (77–100) | Reference |  |
| ***Pre-existing cardiovascular disease*** | Yes | 21 | 7 | 55 (34–90) | 3.10 (1.20–7.90) | <0.05* |
|  | No | 68 | 13 | 88 (80–98) | Reference |  |
| ***Pre-existing chronic pulmonary disease*** | Yes | 12 | 2 | 75 (50–100) | 0.80 (0.20–3.50) | 0.77 |
|  | No | 77 | 18 | 82 (73–93) | Reference |  |
| ***Pre-existing poorly controlled hypertension*** | Yes | 12 | 3 | 89 (71–100) | 1.20 (0.30–4.00) | 0.79 |
|  | No | 77 | 17 | 80 (70–92) | Reference |  |
| ***Pre-existing poorly controlled diabetes*** | Yes | 13 | 3 | 80 (59–100) | 1.30 (0.40–4.50) | 0.68 |
|  | No | 76 | 17 | 82 (72–93) | Reference |  |
| ***Pre-existing peripheral vascular disease*** | Yes | 10 | 3 | 55 (27–100) | 2.80 (0.80–9.80) | 0.10 |
|  | No | 79 | 17 | 84 (75–94) | Reference |  |

BM, bone marrow; CCI, Charlson Comorbidity Index; CI, confidence interval; ECOG PS, Eastern Cooperative Oncology Group Performance Status; ELTS, EUTOS long-term survival; EUTOS, European Treatment and Outcome Study; HR, Hazards Ratio.

* Statistically significant difference (α <0.10) evaluated using univariable Cox proportional hazards regression. P < 0.10 included in multivariable regression model.

^a^ An event is defined as disease progression to accelerated phase or blast crisis, transformation to AML, relapse or death (of any cause), whilst on the TKI or within 60 days off TKI treatment.

^b^ Weight unknown in 15 patients (3 events), BCR-ABL1 transcript type unknown in 23 patients (5 events), BM fibrosis unknown in 42 patients (12 events), BM karyotype abnormalities unknown in 28 patients (9 events), ELTS and Sokal scores unknown in 6 patients (2 events).

^c^ A 10-year increase in age is associated with a 4% increase in the hazard of an event. A 10 kg increase in weight is associated with a 3% increase in the hazard of an event. These effects are not statistically significant.

^d^ Geographic ancestry was assigned using information contained on patient registration forms and in medical records.

^e^ 3 individuals of Middle Eastern/North African ancestry (1 Lebanon, 1 Iran, 1 Egypt) and 3 of Pacific Islander ancestry (Maori).

^f^ Other BCR-ABL1 transcripts: e14a2 with e1a2, e13a2 with e1a2, e1a2, e19a2, and combined e12a2, e14a2 with e1a2.

^g^ Concomitant medicines with the potential for an imatinib drug-drug interaction include CYP3A4 substrates, CYP3A4 inhibitors/inducers, CYP2C8 inhibitors, P-glycoprotein inhibitors, thyroxine, digoxin, paracetamol, antiplatelets and antineoplastic agents.

REFERENCES

1. Patel H, Egorin MJ, Remick SC, et al. Comparison of Child-Pugh (CP) criteria and NCI organ dysfunction working group (NCI-ODWG) criteria for hepatic dysfunction (HD): implications for chemotherapy dosing 2004;22(14_suppl):6051. Accessed September 5, 2020. https://ascopubs.org/doi/abs/10.1200/jco.2004.22.90140.6051

2. Ramanathan RK, Egorin MJ, Takimoto CH, et al. Phase I and pharmacokinetic study of imatinib mesylate in patients with advanced malignancies and varying degrees of liver dysfunction: a study by the National Cancer Institute Organ Dysfunction Working Group. *J Clin Oncol*. 2008;26(4):563-9. doi:10.1200/jco.2007.11.0304

3. Stevens PE, Levin A. Evaluation and management of chronic kidney disease: synopsis of the kidney disease: improving global outcomes 2012 clinical practice guideline. *Ann Intern Med*. Jun 4 2013;158(11):825-30. doi:10.7326/0003-4819-158-11-201306040-00007

4. Colagiuri S, Dickinson S, Girgis S, Colagiuri R. *National evidence based guideline for blood glucose control in type 2 diabetes*. Diabetes Australia and the NHMRC; 2009.

5. National Heart Foundation of Australia. *Guideline for the diagnosis and management of hypertension in adults*. National Heart Foundation of Australia; 2016.

6. Oken MM, Creech RH, Tormey DC, et al. Toxicity and response criteria of the Eastern Cooperative Oncology Group. *Am J Clin Oncol*. Dec 1982;5(6):649-55.

7. Deininger MW, Shah NP, Altman JK, et al. Chronic myeloid leukemia, Version 2.2021, NCCN clinical practice guidelines in oncology. *J Natl Compr Canc Netw*. 2020;18(10):1385-415. doi:10.6004/jnccn.2020.0047

8. Saglio G, Kim DW, Issaragrisil S, et al. Nilotinib versus imatinib for newly diagnosed chronic myeloid leukemia. *N Engl J Med*. Jun 17 2010;362(24):2251-9. doi:10.1056/NEJMoa0912614

9. Kantarjian H, Shah NP, Hochhaus A, et al. Dasatinib versus imatinib in newly diagnosed chronic-phase chronic myeloid leukemia. *N Engl J Med*. Jun 17 2010;362(24):2260-70. doi:10.1056/NEJMoa1002315

10. Kaplan EL, Meier P. Nonparametric estimation from incomplete observations. *J Am Stat Assoc*. 1958;53(282):457-81. doi:10.1080/01621459.1958.10501452

11. Cox DR. Regression models and life-tables. *J R Stat Soc Series B Stat Methodol*. 1972;34(2):187-202. doi:10.1111/j.2517-6161.1972.tb00899.x

12. Chowdhury MZI, Turin TC. Variable selection strategies and its importance in clinical prediction modelling. *Fam Med Community Health*. 2020;8(1):e000262. doi:10.1136/fmch-2019-000262

13. Noordzij M, Leffondre K, van Stralen KJ, Zoccali C, Dekker FW, Jager KJ. When do we need competing risks methods for survival analysis in nephrology? *Nephrol Dial Transplant*. Nov 2013;28(11):2670-7. doi:10.1093/ndt/gft355

14. Austin PC, Lee DS, Fine JP. Introduction to the Analysis of Survival Data in the Presence of Competing Risks. *Circulation*. Feb 9 2016;133(6):601-9. doi:10.1161/circulationaha.115.017719

15. Schmoor C, Bender R, Beyersmann J, Kieser M, Schumacher M. Adverse event development in clinical oncology trials. *Lancet Oncol*. 2016;17(7):e263-4. doi:10.1016/s1470-2045(16)30223-6

16. Fine JP, Gray RJ. A proportional hazards model for the subdistribution of a competing risk. *J Am Stat Assoc*. 1999;94(446):496-509. doi:10.1080/01621459.1999.10474144

17. Scrucca L, Santucci A, Aversa F. Regression modeling of competing risk using R: an in depth guide for clinicians. *Bone Marrow Transplant*. 2010;45(9):1388-95. doi:10.1038/bmt.2009.359

18. Scrucca L, Santucci A, Aversa F. Competing risk analysis using R: an easy guide for clinicians. *Bone Marrow Transplant*. Aug 2007;40(4):381-7. doi:10.1038/sj.bmt.1705727

19. Zhang X, Zhang MJ, Fine J. A proportional hazards regression model for the subdistribution with right-censored and left-truncated competing risks data. *Stat Med*. 2011;30(16):1933-51. doi:10.1002/sim.4264

20. Allignol A, Beyersmann J, Schmoor C. Statistical issues in the analysis of adverse events in time-to-event data. *Pharm Stat*. Jul 2016;15(4):297-305. doi:10.1002/pst.1739

21. Prentice RL, Williams BJ, Peterson AV. On the regression analysis of multivariate failure time data. *Biometrika*. 1981;68(2):373-9. doi:10.2307/2335582
